# A Subcarrier-Pair Based Resource Allocation Scheme Using Proportional Fairness for Cooperative OFDM-Based Cognitive Radio Networks

**DOI:** 10.3390/s130810306

**Published:** 2013-08-09

**Authors:** Yongtao Ma, Liuji Zhou, Kaihua Liu

**Affiliations:** School of Electronic Information Engineering, Tianjin University, 92 Weijin Road, Nankai District, Tianjin 300072, China; E-Mails: mayongtao@tju.edu.cn (Y.M.); liukaihua@tju.edu.cn (K.L.)

**Keywords:** cognitive radio, cooperative communication, resource allocation, proportional fairness, spectrum sharing

## Abstract

The paper presents a joint subcarrier-pair based resource allocation algorithm in order to improve the efficiency and fairness of cooperative multiuser orthogonal frequency division multiplexing (MU-OFDM) cognitive radio (CR) systems. A communication model where one source node communicates with one destination node assisted by one half-duplex decode-and-forward (DF) relay is considered in the paper. An interference-limited environment is considered, with the constraint of transmitted sum-power over all channels and aggregate average interference towards multiple primary users (PUs). The proposed resource allocation algorithm is capable of maximizing both the system transmission efficiency and fairness among secondary users (SUs). Besides, the proposed algorithm can also keep the interference introduced to the PU bands below a threshold. A proportional fairness constraint is used to assure that each SU can achieve a required data rate, with quality of service guarantees. Moreover, we extend the analysis to the scenario where each cooperative SU has no channel state information (CSI) about non-adjacent links. We analyzed the throughput and fairness tradeoff in CR system. A detailed analysis of the performance of the proposed algorithm is presented with the simulation results.

## 1. Introduction

Cognitive radio technology (CR) has been proposed as a relatively new concept for improving the overall utilization of spectrum bands. This promising technology can allow the unlicensed secondary users (SUs, also referred to as CR users or CRUs) to access those frequency bands which are not currently being used by licensed primary users (PUs) in a given geographical area [1,2]. Cooperative communication technology [3] allows network nodes with single antennas to use other network nodes' antennas to transmit data, which can generate a virtual multiple-input multiple-output (MIMO) system. Cooperative spectrum sensing is a viable sensing technique to enhance spectral utilization efficiency of secondary users while ensuring the quality of service (QoS) of primary users [4]. In a CR network, SUs are allowed to transmit over the frequency bands of PUs as long as the resulting aggregate interference is kept below a certain threshold. This threshold is known as interference temperature constraint or interference power constraint [1]. As SUs can design power and subcarrier allocation strategies subject to such interference power constraints, the interference introduced to PUs is effectively controlled. A great deal of resource allocation algorithms and interference control strategies has been proposed for spectrum-sharing CR networks. For example, the optimal power allocation strategies to maximize the transmitted data rate of the secondary user with an effective protection of the primary user were studied in [5,6] for spectrum-sharing CR networks.

Orthogonal frequency division multiplexing (OFDM) is an attractive modulation scheme for users in a CR system due to its flexibility in allocating resources among SUs. Since both SUs and PUs may exist in side-by-side bands, yet have different access technologies, mutual interference is the limiting factor for the performance of both networks. Thus, using of the classical subcarrier allocation and power loading algorithms, such as uniform power but variable rate and water-filling algorithms maximizing the transmission capacity of an OFDM-based conventional wireless network may result in higher mutual interference in the PUs' band. There is only one group of users in such a wireless network, *i.e.*, PUs, for a CR system.

According to the latest literature on resource allocation in cooperative communication [7–17], the relay users in the system do not transmit their own data and merely help other non-relay users transmit data. In some wireless applications such as cellular networks, however, each user has its own data to transmit so that it should allocate its total constrained power and subcarriers properly in transmitting its own data and relaying other users' data [18,19]. Tourki [19] focused on efficiency issues by studying how to maximize the total transmitted data rate in non-orthogonal amplify-and-forward (AF) cooperative scheme, which ignores the fairness among the cooperative users. According to [20,21], equal power allocation (EPA) among subcarriers was proposed to separate the user selection from the power of subcarrier. With EPA, the EPA-PRG (proportional rate greedy) [22] algorithm is proposed to maximize the system throughput while keeping the fairness. However, cooperative transmission technology isn't applied in this algorithm. In [23], a linear water-filling scheme (LWF-PI) was proposed. This algorithm maximized the overall transmitted data rate of the CR system while keeping the interference introduced to the PU bands below a threshold. However, the fairness among users was ignored. Chandrashekar *et al.* [24] proposed an algorithm which is capable of maximizing the total transmitted data rate and achieving a high proportional fairness index. However, this algorithm cannot be applied to the CR network where we must adjust the interference introduced to the PU bands below a threshold. Tan [25] proposed a joint subcarrier and power algorithm based on Blotto games. This algorithm can achieve a good trade-off performance between fairness and efficiency in OFDMA-based cognitive radio network (CRN), but it cannot obtain the effectiveness of multiuser diversity for the SUs without ability to generate a virtual MIMO system.

A novel scheme was presented in [26] for the allocation of subcarriers, rates, and power in orthogonal frequency-division multiple-access (OFDMA) networks. The resource-allocation problem was solved by decomposing it into a hierarchy of sub-problems. A joint subcarrier and power allocation algorithm was presented in [27] for cooperative MU-OFDM CR systems. In [28], a survey of resource allocation and scheduling schemes in OFDMA wireless networks was presented. Nader *et al.* in [29] considered the practical case in which only partial CSI for the wireless channel between the secondary base station and SUs is available at the secondary base station. They formulated the resource allocation problem in the secondary network as an optimization problem in which the objective was to maximize the weighted sum rate of the secondary users. A novel sub-channel and transmission power allocation scheme was proposed in [30] for multi-cell OFDMA networks with CR functionality. Tianxiang *et al.* in [31] discussed optimization over the relay assignment, subcarrier allocation, per node power control, and heterogeneous quality-of-service (QoS) provisioning. Sabit *et al.* in [32] investigated the performance of an OFDM-based CR spectrum sharing communication system that assumed random allocation and absence of the PU channel occupation information. Hong Xu *et al.* in [33] formulated a unifying optimization framework based on Nash bargaining solutions to fairly and efficiently allocate resources between primary and secondary networks, in both decentralized and centralized settings. As the optimal resource allocation scheme was highly complex, G. B. *et al.* [34] proposed a low complexity suboptimal subcarrier and power allocation scheme. They also proposed a suboptimal subcarrier allocation scheme that can guarantee a certain level of fairness among CR users. Naeem *et al.* introduced in [35] a hybrid heuristic algorithm for the relay assignment and power allocation problem which is a non-convex mixed-integer non-linear optimization problem, and this problem is generally non-deterministic polynomial-time (NP)-hard.

In this paper, a joint subcarrier-pair based resource allocation algorithm in order to improve both efficiency and fairness index is presented first. The definition of fairness is borrowed from the networking literature. In contrast with [36], where large channel fluctuations are intentionally created with “dumb” antennas for long-term proportional fairness resource allocation, this paper proposes a subcarrier-pair based resource allocation algorithm to maintain proportional rates among SUs for each channel realization, which ensures the rates of different SUs to be proportional in any time scale of interest. By formulating the resource allocation and pairing problem in this way, it will be shown that a high transmitted data rate for all SUs (even those with poor channel gains) can be achieved with low computational complexity. Moreover, we extend the analysis to the case in which each SU can only have access to CSI of its adjacent links. This is a more realistic scenario when network nodes are mobile and the timely CSI cannot be exchanged between cooperative users. Consequently, each user can only have access to statistical CSI of non-adjacent links. It is shown that the system performance deteriorates due to limited CSI but still outperforms that of equal power allocation scheme. The key contributions of this work are:

1. It is considered that SUs need to transmit their own data directly to the destination, and in the next phase they also help their partner forward the data received in previous phase to the destination. Simulation results show that in the same situations the system transmitted data rate by proposed algorithm is the highest than that by LWF-PI algorithm [23], EPA algorithm [21] and the Optimal Scheme [37].
2. The proposed subcarrier-pair based resource allocation algorithm ensures the rates of different SUs to be proportional in any time scale of interest, simulation results shown that a high transmitted data rate for all SUs (even those with poor channel gains) can be achieved.
3. It is considered that SU has no CSI about non-adjacent link. In this case, we take full advantage of the statistical information of the non-adjacent links.

Notation: In this paper, a variable with “underline” ■̲ denotes the temporary optimal values within each iteration process, the “double underline” ■̳ denotes the optimal value, and the “bar” ■̄ denotes the statistical average value. *E* (■) denotes the expectation operator, and $\overset{︷}{■}$ denotes the optimal value when only partial CSI can be obtained by SUs.

## 2. System Model and Problem Formulation

**Figure 1.**
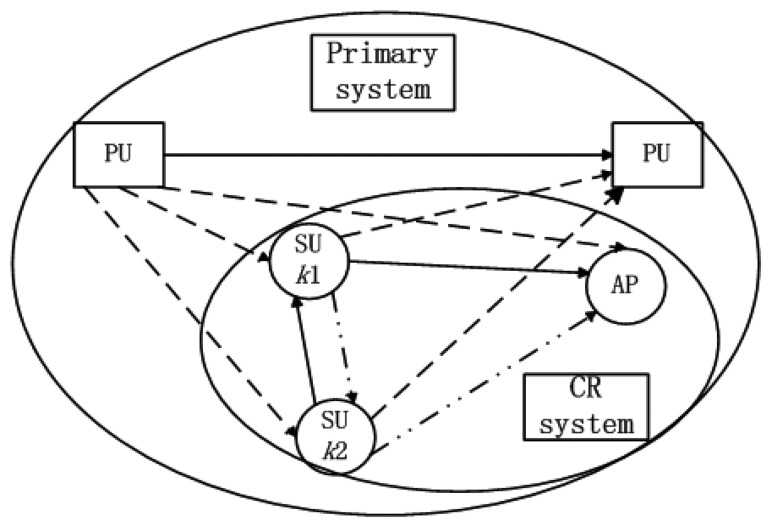
A cooperative MU-OFDM CR uplink system.

We consider a hybrid network consisting of a primary network (PRN) and a cognitive radio network (CRN) as shown in Figure 1. The CRN consists of a CR access point (AP) and 2*K* SUs. The PRN and CRN co-exist within the same geographical area. The access mechanism/modulation format in SUs' band is OFDM. Our focus is mainly on the uplink radio resource allocation in the CRN. The SUs are trying to find the opportunity to access to the AP.

**Figure 2.**
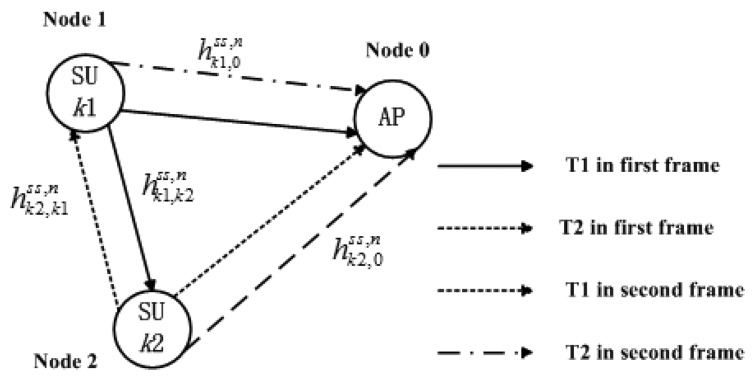
Model for cooperative transmission.

**Figure 3.**
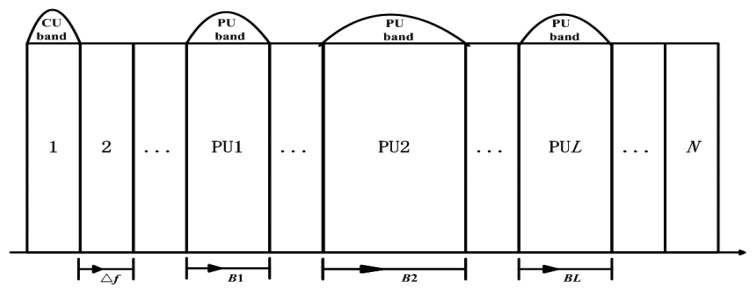
spectrum access model of cognitive radio system.

According to [37,38], we also consider that the frequency bands of bandwidth *B*_1_, *B*_2_, …, *B_L_* which have been occupied by *L* PUs are sensed by the CR system and known to SU transmitters. Every two SUs form a cooperative partner and they are relay node for each other. As shown in Figure 2, the *k*th (1 ≤ *k* ≤ *K*) cooperative partner consists of two SU transmitters, *k*1 and *k*2. As is assumed in [23,37,38], we consider the same side-by-side CR radio access model. The unoccupied bandwidth sensed by SUs for opportunistic spectrum access is located on each side of *L* PU bands as shown in Figure 3. The available bandwidth for CR transmission is divided into *N* subcarriers based on OFDM system. It is considered that the access mechanism/modulation format in PUs' band is not known to the CR system and the bandwidth for each CR subcarriers is Δ*f Hz*. Some symbols are shown in Table 1.

In general, there are three instantaneous fading gains in the uplink transmission scenario shown in Figure 1:

(1) The gains between the SU's transmitter and SU's receiver or AP for the *n*th subcarrier denoted as $h_{ki,kj}^{ss,n}$, $h_{ki,0}^{ss,n}$, respectively.
(2) The gains between the SU's transmitter and *l*th PU's receiver, denoted as $h_{ki,pl}^{sp,n}$.
(3) The gains between the *l*th PU's transmitter and the SU's receiver or AP, denoted as $h_{pl,ki}^{ps,n}$, $h_{pl,0}^{ps,n}$, respectively.

The channel gains are modeled as independent zero-mean complex Gaussian random variables, where *ki* denotes the *i*th SU in *k*th cooperation partner and *pl* denotes the *l*th PU band. According to [39], it is considered that these instantaneous fading gains are perfectly known at the SU's transmitter. Specifically, we assume that the SU's receiver can estimate channel gains $h_{ki,kj}^{ss,n}$ and $h_{pl,ki}^{ps,n}$ and report to the CR transmitter. In Section 3.2, we will study the case where the instantaneous fading gains of the non-adjacent links are not perfectly known at the SU transmitter but the statistical information of the non-adjacent links are known at the SU transmitter. Moreover, it is assumed that primary receiver can estimate the channel $h_{ki,pl}^{sp,n}$ which is reported to the SU transmitter through a common control channel.

### 2.1. Cooperative Transmission among SUs

The scenario of a three-node DF diversity model is considered, where one source communicates with one destination assisted by one half-duplex relay, as shown in Figure 2. One transmission period is divided into two consecutive frames. Communication takes place in two phases (listening phase T1 and relaying phase T2, the definition is according to the working state of relay user) for each frame. The power allocation scheme for *k*th cooperative partner on subcarrier *n* is shown in Table 2. The source node broadcasts its signal to relay and AP in T1, whereas the relay and AP listen. The relay decodes the signal and forwards it to AP in T2. It is denoted that the subcarrier *n* in T1 is pairing with subcarrier SP1(*n*) in T2 for first frame, and pairing with subcarrier SP2(*n*) for second frame. In the first frame, *k*2th SU receives data in this time slot while *k*1th SU transmits a symbol *x*_k1_(*t*) with power level $P_{k1,1}^{n,\text{SP}2(n)}$ on *n*th subcarrier in T1. The symbol is received by node 0 (AP) and overheard by *k*2th SU as:

(1) $$\begin{array}{l} y_{k1,0}(t)=h_{k1,0}^{ss,n}\sqrt{P_{k1,1}^{n,\text{SP}1(n)}}x_{k1}(t)+z_{0}^{\left(1\right)}(t)+\vartheta_{0}^{\left(1\right)}(t)\\ y_{k1,k2}(t)=h_{k1,k2}^{ss,n}\sqrt{P_{k1,1}^{n,\text{SP}1(n)}}x_{k1}(t)+z_{k2}^{\left(1\right)}(t)+\vartheta_{k2}^{\left(1\right)}(t) \end{array}$$

During this interval, the *k*2th SU decodes its overheard signal as $x_{k1}^{*}(t)$ and transmits it to the AP on SP1(*n*) subcarrier in T2 with the power level $P_{k2,1}^{n,\text{SP}2(n)}$. Then the AP receives the signal as:

(2) $$y_{k2,0}(t)=h_{k2,0}^{ss,\text{SP}1(n)}\sqrt{P_{k2,1}^{n,\text{SP}1(n)}}x_{k1}^{*}(t)+z_{0}^{\left(2\right)}(t)+\vartheta_{0}^{\left(2\right)}(t)$$

In the second frame, the roles of *k*1th SU and k2th SU are reversed. Similarly, *k*2th SU transmits a symbol *x_k_*_2_ (*t*) with power level $P_{k2,2}^{n,\text{SP}2(n)}$ on the nth subcarrier in T1. The symbol is received by node 0 (AP) and overheard by *k*1th SU as:

(3) $$\begin{array}{l} y_{k2,0}(t)=h_{k2,0}^{ss,n}\sqrt{P_{k2,2}^{n,\text{SP}2(n)}}x_{k2}(t)+z_{0}^{\left(3\right)}(t)+\vartheta_{0}^{\left(3\right)}(t)\\ y_{k2,k1}(t)=h_{k2,k1}^{ss,n}\sqrt{P_{k2,2}^{n,\text{SP}2(n)}}x_{k2}(t)+z_{k1}^{\left(3\right)}(t)+\vartheta_{k1}^{\left(3\right)}(t) \end{array}$$

In T2 of second frame, the AP node receives the noisy signal which is relayed by *k*1th SU with the power level $P_{k1,2}^{n,\text{SP}2(n)}$, *i.e.*:

(4) $$y_{k1,0}(t)=h_{k1,0}^{ss,\text{SP}2(n)}\sqrt{P_{k1,2}^{n,\text{SP}2(n)}}x_{k2}^{*}(t)+z_{0}^{\left(4\right)}(t)+\vartheta_{0}^{\left(4\right)}(t)$$

### 2.2. Mutual Interference between PU Bands and CR Users

In the MU-OFDM CR system, due to the coexistence of PUs and SUs in side by side bands, it is necessary to consider the mutual interference between PUs and SUs. There are two types of interference in the system. One is introduced by the PUs into the SUs band, and the other is introduced by the SUs into the PUs' band. In what follows, we provide brief description and mathematical models for interference between SUs and PUs.

#### 2.2.1. The Interference Introduced into PUs by SUs

CR interference is introduced into the PU spectrum by CR out-of-band (OOB) emissions. OOB emissions arise as a result of transmit pulse shaping such that a portion of the CR radiated power in a vacant subcarrier is leaked into neighboring bands occupied by the PUs. According to [23], the interference factor which is the integration of the power density spectrum of the *n*th subcarrier across the *l*th PU band, and can be written as:

(5) $$S_{ki,pl}^{n}(d_{nl})={\left|h_{ki,pl}^{sp,n}\right|}^{2}Ts\int_{d_{nl}-\frac{B_{l}}{2}}^{d_{nl}+\frac{B_{l}}{2}}{\left(\frac{\sin(\pi fTs)}{\pi fTs}\right)}^{2}df$$

where *Ts* denotes the symbol duration, *d*_nl_ denotes the distance in frequency between the *n*th subcarriers of SU band and *l*th PU band, and *B*_l_ represents occupied bandwidth by *l*th PU. It can be shown from Equation (5) that the interference to PU band is related to the distance between SU band and PU band.

#### 2.2.2. The Interference Introduced into SUs by PUs

The interference introduced into *ki*th SU and AP node transmitting in *n*th subcarrier by *l*th PU can be denoted as $J_{pl,ki}^{n}$, $J_{pl,0}^{n}$, respectively. According to [37], the interference value $J_{pl,ki}^{n}$, $J_{pl,0}^{n}$ can be written as:

(6) $$\begin{array}{l} J_{pl,ki}^{n}(d_{nl},P_{PU})={\left|h_{pl,ki}^{sp,n}\right|}^{2}\int_{d_{nl}-\frac{\Delta f}{2}}^{d_{nl}+\frac{\Delta f}{2}}E\{I_{N}(w,\phi_{PU}(e^{jw}))\}dw\\ J_{pl,0}^{n}(d_{nl},P_{PU})={\left|h_{pl,0}^{sp,n}\right|}^{2}\int_{d_{nl}-\frac{\Delta f}{2}}^{d_{nl}+\frac{\Delta f}{2}}E\{I_{N}(w,\phi_{PU}(e^{jw}))\}dw\\ E\{I_{N}(w,\phi_{PU}(e^{jw})\}=\frac{1}{2\pi M}\int_{-\pi }^{\pi }\phi_{PU}(e^{jw}){\left(\frac{\sin(w-\varsigma )M/2}{\sin(w-\varsigma )/2}\right)}^{2}d\varsigma \end{array}$$

where *w* represents the frequency normalized to the sampling frequency, E{I_N_(·)}is the power density spectrum of the PU signal after *M*-fast Fourier transform (FFT) processing, *φ_PU_* (*e^jw^*) is the power density spectrum of the PU signal. The PU signal has been taken to be an elliptically filtered white noise process with amplitude *P_PU_*.

According to [40], using a relay is advantageous when:

(7) $$\begin{array}{l} \text{frame}1:\min(\frac{{\left|h_{k1,k2}^{ss,n}\right|}^{2}}{\sigma^{2}+\overset{L}{\underset{l=1}{\text{∑}}}J_{pl,k2}^{n}},\frac{{\left|h_{k2,0}^{ss,\text{SP}1(n)}\right|}^{2}}{\sigma^{2}+\overset{L}{\underset{l=1}{\text{∑}}}J_{pl,0}^{\text{SP}1(n)}})\geq \frac{{\left|h_{k1,0}^{ss,n}\right|}^{2}}{\sigma^{2}+\overset{L}{\underset{l=1}{\text{∑}}}J_{pl,0}^{n}}\\ \text{frame}2:\min(\frac{{\left|h_{k2,k1}^{ss,n}\right|}^{2}}{\sigma^{2}+\overset{L}{\underset{l=1}{\text{∑}}}J_{pl,k1}^{n}},\frac{{\left|h_{k1,0}^{ss,\text{SP}2(n)}\right|}^{2}}{\sigma^{2}\text{+}\overset{L}{\underset{l=1}{\text{∑}}}J_{pl,0}^{\text{SP}2(n)}})\geq \frac{{\left|h_{k2,0}^{ss,n}\right|}^{2}}{\sigma^{2}+\overset{L}{\underset{l=1}{\text{∑}}}J_{pl,0}^{n}} \end{array}$$

in selective DF mode. It is considered that the link of source node→ relay node and link of relay node→ destination node are better than that of source node→ destination node. Otherwise, the relay keeps idle on subcarrier *n* in the relaying phase for $x_{k2}^{*}(t)$ or $x_{k1}^{*}(t)$. In this paper, we just consider the case in which the relays keep working on each subcarrier, *i.e.*, the Equation (7) is always true.

## 3. Optimization Problem Formulation

In this section, we analyze the joint optimization of subcarrier-pair based resource allocation algorithm for OFDM-DF based on full CSI and partial CSI, respectively. We are interested in how each SU allocates its power properly across its own data and its relayed data so as to maximize the system transmitted data rate while maintaining reasonable fairness between SUs. The optimization problem is formulated firstly and then solved in the dual domain. It is assumed that the PUs have a constant-rate, constant-power transmission, while the SUs are capable to adjust transmit power over different fading states based on the CSI of the CR network. We study a type of constraint imposed over the secondary transmission to protect the PUs by limiting the interference introduced to the PUs below a threshold.

### 3.1. Resource Allocation and Subcarrier Pairing Scheme Based on the OFDM-DF

The CR AP combines the received signals from the source node in T1 and the relay node in T2 through the maximal ratio combining. The transmit power is adjusted in each SU's transmitter. According to [27] and [41], when the link of source node->relay node transmission is successful for entire DF process, the transmission rate of *k1*th SU and *k2*th SU at *n* subcarrier in relaying mode, which is connected via the Shannon capacity formula, can be shown as $I_{k1}^{n,\text{SP}1(n)}$ and $I_{k2}^{n,\text{SP}2(n)}$, respectively:

(8) $$\begin{array}{l} I_{k1}^{n,\text{SP}1(n)}=\frac{\Delta f}{4}\log_{2}\{1+\min({\left|h_{k1,k2}^{ss,n}\right|}^{2}\frac{P_{k1,1}^{n,\text{SP}1(n)}}{\sigma^{2}+\overset{L}{\underset{l=1}{\text{∑}}}J_{pl,k2}^{n}},{\left|h_{k1,0}^{ss,n}\right|}^{2}\frac{P_{k1,1}^{n,\text{SP}1(n)}}{\sigma^{2}+\overset{L}{\underset{l=1}{\text{∑}}}J_{pl,0}^{n}}+{\left|h_{k2,0}^{ss,\text{SP}1(n)}\right|}^{2}\frac{P_{k2,1}^{n,\text{SP}1(n)}}{\sigma^{2}+\overset{L}{\underset{l=1}{\text{∑}}}J_{pl,0}^{\text{SP}1(n)}})\}\\ I_{k2}^{n,\text{SP}2(n)}=\frac{\Delta f}{4}\log_{2}\{1+\min({\left|h_{k2,k1}^{ss,n}\right|}^{2}\frac{P_{k2,2}^{n,\text{SP}2(n)}}{\sigma^{2}+\overset{L}{\underset{l=1}{\text{∑}}}J_{pl,k1}^{n}},{\left|h_{k2,0}^{ss,n}\right|}^{2}\frac{P_{k2,2}^{n,\text{SP}2(n)}}{\sigma^{2}+\overset{L}{\underset{l=1}{\text{∑}}}J_{pl,0}^{n}}+{\left|h_{k1,0}^{ss,\text{SP}2(n)}\right|}^{2}\frac{P_{k1,2}^{n,\text{SP}2(n)}}{\sigma^{2}+\overset{L}{\underset{l=1}{\text{∑}}}J_{pl,0}^{\text{SP}2(n)}})\} \end{array}$$

where *σ*^2^ denotes the Additive White Gaussian Noise(AWGN) variance. Here, it is assumed that all the channel gains are constant during two frames and the link between cooperative partners are symmetric, *i.e.*, $h_{k1,k2}^{ss,n}=h_{k2,k1}^{ss,n}$ for all *k*. The factor 1/4 in Equation (8) results from the fact that the transmission takes four slots in the cooperative scheme.

Let $P_{k1,i}^{n,\text{SPi}(n)}+P_{k2,i}^{n,\text{SPi}(n)}=P_{k1}^{n,\text{SPi}(n)}$, *i* = 1,2 where *i* denotes the *i*th frame. This formula means that the average of the transmit power of the source node $P_{k1,i}^{n,\text{SP}i(n)}$ and that of the relay node $P_{k2,i}^{n,\text{SP}i(n)}$ is constrained to be $P_{ki}^{n,\text{SP}i(n)}$, which is the allocated power on subcarrier *n* at the source node for direct transmission. According to [42], the solution to this problem is the transmitted data rate and it is maximized when:

(9) $$\begin{array}{l} {\left|h_{k1,k2}^{ss,n}\right|}^{2}\frac{P_{k1,1}^{n,\text{SP}1(n)}}{\sigma^{2}+\overset{L}{\underset{l=1}{\text{∑}}}J_{pl,k2}^{n}}={\left|h_{k1,0}^{ss,n}\right|}^{2}\frac{P_{k1,1}^{n,\text{SP}1(n)}}{\sigma^{2}+\overset{L}{\underset{l=1}{\text{∑}}}J_{pl,0}^{n}}+{\left|h_{k2,0}^{ss,\text{SP}1(n)}\right|}^{2}\frac{P_{k2,1}^{n,\text{SP}1(n)}}{\sigma^{2}+\overset{L}{\underset{l=1}{\text{∑}}}J_{pl,0}^{\text{SP}1(n)}}\\ {\left|h_{k2,k1}^{ss,n}\right|}^{2}\frac{P_{k2,2}^{n,\text{SP}2(n)}}{\sigma^{2}+\overset{L}{\underset{l=1}{\text{∑}}}J_{pl,k1}^{n}}={\left|h_{k2,0}^{ss,n}\right|}^{2}\frac{P_{k2,2}^{n,\text{SP}2(n)}}{\sigma^{2}+\overset{L}{\underset{l=1}{\text{∑}}}J_{pl,0}^{n}}+{\left|h_{k1,0}^{ss,\text{SP}2(n)}\right|}^{2}\frac{P_{k1,2}^{n,\text{SP}2(n)}}{\sigma^{2}+\overset{L}{\underset{l=1}{\text{∑}}}J_{pl,0}^{\text{SP}2(n)}}\\ \text{for all}1\leq n\leq N,1\leq k\leq K \end{array}$$

Let:

(10) $$\gamma_{k1,k2}^{n}=\frac{{\left|h_{k1,k2}^{ss,n}\right|}^{2}}{\sigma^{2}+\overset{L}{\underset{l=1}{\text{∑}}}J_{pl,k2}^{n}},\gamma_{k1,0}^{n}=\frac{{\left|h_{k1,0}^{ss,n}\right|}^{2}}{\sigma^{2}+\overset{L}{\underset{l=1}{\text{∑}}}J_{pl,0}^{n}},\gamma_{k2,0}^{n}=\frac{{\left|h_{k2,0}^{ss,n}\right|}^{2}}{\sigma^{2}+\overset{L}{\underset{l=1}{\text{∑}}}J_{pl,0}^{n}},\gamma_{k2,k1}^{n}=\frac{{\left|h_{k2,k1}^{ss,n}\right|}^{2}}{\sigma^{2}+\overset{L}{\underset{l=1}{\text{∑}}}J_{pl,k1}^{n}}$$

Together with $P_{k1,i}^{n,\text{SP}i(n)}+P_{k2,i}^{n,\text{SP}i(n)}=P_{k,i}^{n,\text{SP}i(n)}$, *i* = 1, 2 we can obtain that:

(11) $$\begin{array}{l} P_{k1,1}^{n,\text{SP}1(n)}=\frac{\gamma_{k2,0}^{\text{SP}1(n)}}{\gamma_{k1,k2}^{n}-\gamma_{k1,0}^{n}+\gamma_{k2,0}^{\text{SP}1(n)}}P_{k1}^{n,\text{SP}1(n)},P_{k2,1}^{n,\text{SP}1(n)}=\frac{\gamma_{k1,k2}^{n}-\gamma_{k1,0}^{n}}{\gamma_{k1,k2}^{n}-\gamma_{k1,0}^{n}+\gamma_{k2,0}^{\text{SP}1(n)}}P_{k1}^{n,\text{SP}1(n)}\\ P_{k1,2}^{n,\text{SP}2(n)}=\frac{\gamma_{k2,k1}^{n}-\gamma_{k2,0}^{n}}{\gamma_{k2,k1}^{n}-\gamma_{k2,0}^{n}+\gamma_{k1,0}^{\text{SP}2(n)}}P_{k2}^{n,\text{SP}2(n)},P_{k2,2}^{n,\text{SP}2(n)}=\frac{\gamma_{k1,0}^{\text{SP}2(n)}}{\gamma_{k2,k1}^{n}-\gamma_{k2,0}^{n}+\gamma_{k1,0}^{\text{SP}2(n)}}P_{k2}^{n,\text{SP}2(n)} \end{array}$$

Denote $\eta_{k1}^{n,\text{SP}1(n)}$, $\eta_{k2}^{n,\text{SP}2(n)}$ as the equivalent channel gain given by:

(12) $$\eta_{k1}^{n,\text{SP}1(n)}=\frac{\gamma_{k1,k2}^{n}\gamma_{k2,0}^{\text{SP}1(n)}}{\gamma_{k1,k2}^{n}-\gamma_{k1,0}^{n}+\gamma_{k2,0}^{\text{SP}1(n)}},\eta_{k2}^{n,\text{SP}2(n)}=\frac{\gamma_{k2,k1}^{n}\gamma_{k1,0}^{\text{SP}2(n)}}{\gamma_{k2,k1}^{n}-\gamma_{k2,0}^{n}+\gamma_{k1,0}^{\text{SP}2(n)}}(12)$$

By now, we can unify the transmitted data rate as:

(13) $$\begin{array}{c} I_{k1}^{n,\text{SP}1(n)}(P_{k1}^{n,\text{SP}1(n)})=\frac{\Delta f}{4}\log_{2}\{1+\eta_{k1}^{n,\text{SP}1(n)}P_{k1}^{n,\text{SP}1(n)}\}\\ I_{k2}^{n,\text{SP}2(n)}(P_{k2}^{n,\text{SP}2(n)})=\frac{\Delta f}{4}\log_{2}\{1+\eta_{k2}^{n,\text{SP}2(n)}P_{k2}^{n,\text{SP}2(n)}\} \end{array}$$

The proportional fairness is used as the optimized objective to develop subcarrier-pair based resource allocation algorithm in order to maintain a balance between system efficiency and fairness. According to [43,44] and the theorem in [45], we can readily deduce that there exists one unique proportionally fair allocation which can be attained by maximizing the objective function $\Psi =\overset{K}{\underset{k=1}{\text{∑}}}\overset{N}{\underset{n=1}{\text{∑}}}\omega_{k}\rho_{kn}(I_{k1}^{n,\text{SP}1(n)}+I_{k2}^{n,\text{SP}2(n)})$ over the feasible set, where *ω_k_* is the weighting factor to make the *K* cooperative partners achieve the desirable transmitted data rate. Besides, we should keep the instantaneous interference introduced to the PUs below a certain threshold. The constraints include the aspects of satisfying the maximum power and interference constraints as well as the minimum rate requirements. Therefore, the resource allocation problem can be formulated mathematically as given in Equation (14). Constraint C1 corresponds to the subcarrier allocation constraint that each subcarrier *n* only can be allocated to one cooperative partner. C2 and C3 define that the sum of all the transmission powers of a particular SU on different subcarriers can't be greater than the maximum allowed limit for that particular SU. C4 ensures the cumulative interference from all SUs and through all subcarriers on a particular PU should not be greater than the interference limit set. C5 ensure that each SU can obtain the minimum rate requirements. This constraint precludes the possibility of multiple SUs simultaneously transmitting at the same subcarrier:

(14) $$\begin{array}{l} \max\Psi =\overset{K}{\underset{k=1}{\text{∑}}}\overset{N}{\underset{n=1}{\text{∑}}}\omega_{k}\rho_{kn}(I_{k1}^{n,\text{SP}1(n)}+I_{k2}^{n,\text{SP}2(n)})\\ \text{subject to C}1:\overset{K}{\underset{k=1}{\text{∑}}}\rho_{kn}\leq 1,\rho_{kn}\in \{0,1\},\forall_{n}\\ \text{C}2:\overset{N}{\underset{n=1}{\text{∑}}}\rho_{kn}P_{k1}^{n,\text{SP}1(n)}\leq \frac{P_{t}}{2},\text{C3}:\overset{N}{\underset{n=1}{\text{∑}}}\rho_{kn}P_{k2}^{n,\text{SP}2(n)}\leq \frac{P_{t}}{2}\\ \text{C4}:\overset{K}{\underset{k=1}{\text{∑}}}\overset{N}{\underset{n=1}{\text{∑}}}\rho_{kn}[P_{k1,1}^{n,\text{SP}1(n)}S_{k1,pl}^{n}+P_{k2,2}^{n,\text{SP}2(n)}S_{k2,pl}^{n}+P_{k1,2}^{n,\text{SP}2(n)}S_{k1,pl}^{\text{SP}2(n)}+P_{k2,1}^{n,\text{SP}1(n)}S_{k2,pl}^{\text{SP}1(n)}]\leq Ith^{\left(l\right)}\\ \text{C}5:\overset{N}{\underset{n=1}{\text{∑}}}\rho_{kn}\{I_{k1}^{n,\text{SP}1(n)}+I_{k2}^{n,\text{SP}2(n)}\}\geq R_{k}\\ \text{for all}\;l=\{1,2,\ldots ,L\},k=\{1,2,\ldots ,k\} \end{array}$$

where *K* denotes the number of cooperative partner, *k* denotes the *k*th cooperative partner, *L* denotes the number of PUs, *l* denotes the *l*th PU, ρ*_kn_* is the allocation indicator that equals 1 when the *n*th subcarrier is allocated to the *k*th cooperative partner and 0 otherwise, *P_i_* is the transmit power budget for each cooperative partner, the factor of 1/2 for the terms of *P_t_*/2 which results from the fact that it is a normalization for the transmissions within the duration of a frame, *Ith*^(*l*)^ denotes the maximum allowable interference level at the *l*th PU receiver, *R_k_* is the minimum transmitted data rate for *k*th cooperative partners.

The optimal solution to Equation (14) can be found by performing an exhaustive search with computational complexity *O*(*K^N^Z*) [46], where *K^N^* is the number of possible subcarrier allocations and *Z* is the complexity of a power allocation algorithm for each subcarrier allocation. To reduce the exponential computational complexity, a suboptimum resource allocation algorithm with less computational complexity is developed in the following. The dual decomposition approach is used to solve the problem. The dual problem of Equation (14) can be formulated as:

(15) $$\begin{array}{l} \underset{\begin{array}{l} \lambda^{\left(0\right)},\lambda^{\left(1\right)},\\ \lambda^{\left(2\right)},\lambda^{\left(3\right)},\lambda^{\left(4\right)} \end{array}}{\min}\underset{\text{P},\text{ρ}}{\max}\Psi =\overset{K}{\underset{k=1}{\text{∑}}}\overset{N}{\underset{n=1}{\text{∑}}}\omega_{k}\rho_{kn}(I_{k1}^{n,\text{SP}1(n)}+I_{k2}^{n,\text{SP}2(n)})+\overset{N}{\underset{n=1}{\text{∑}}}\lambda_{n}^{\left(0\right)}(1-\overset{K}{\underset{k=1}{\text{∑}}}\rho_{kn})+\overset{K}{\underset{k=1}{\text{∑}}}\lambda_{k}^{\left(1\right)}\{\frac{P_{t}}{2}-\overset{N}{\underset{n=1}{\text{∑}}}\rho_{kn}P_{k1}^{n,S}\\ +\overset{K}{\underset{k=1}{\text{∑}}}\lambda_{k}^{\left(2\right)}\{\frac{P_{t}}{2}-\overset{N}{\underset{n=1}{\text{∑}}}\rho_{kn}P_{k2}^{n,SP2(n)}\}+\overset{L}{\underset{l=1}{\text{∑}}}\lambda_{l}^{\left(3\right)}\{Ith^{\left(l\right)}-\overset{K}{\underset{n=1}{\text{∑}}}\overset{N}{\underset{n=1}{\text{∑}}}\rho_{kn}[P_{k1,1}^{n,\text{SP}1(n)}S_{k1,pl}^{n}+P_{k2,2}^{n,\text{SP}2(n)}S_{k2,pl}^{n}\\ +P_{k1,2}^{n,\text{SP}2(n)}S_{k1,pl}^{\text{SP}2(n)}+P_{k2,1}^{n,\text{SP}1(n)}S_{k2,pl}^{\text{SP}1(n)}]\}+\overset{K}{\underset{k=1}{\text{∑}}}\lambda_{k}^{\left(4\right)}[\overset{N}{\underset{n=1}{\text{∑}}}\rho_{kn}(I_{k1}^{n,\text{SP}1(n)}+I_{k2}^{n,\text{SP}2(n)}-R_{k})]\\ \text{Subject to}\;P_{ki,j}^{n,\text{SP}i(n)}\geq 0,\lambda =\{\lambda_{n}^{\left(0\right)},\lambda_{k}^{\left(1\right)},\lambda_{l}^{\left(2\right)},\lambda_{k}^{\left(3\right)},\lambda_{k}^{\left(4\right)}\}\geq 0\\ n\in \{1,2,\ldots ,N\},\{i,j\}\in \{1,2\},k\in \{1,2\ldots ,K\} \end{array}$$

where *i* and *j* denote the *i*th and *j*th SU of the *k*th cooperative partner, respectively. The values of $\left\{\lambda_{n}^{\left(0\right)},\lambda_{k}^{\left(1\right)},\lambda_{k}^{\left(2\right)},\lambda_{l}^{\left(3\right)},\lambda_{k}^{\left(4\right)}\right\}$ are the introduced Lagrange multipliers. In the future using, we denote a vector shown in Equation (16):

(16) $$\begin{array}{lll} \lambda^{\left(0\right)}={\left[\lambda_{1}^{\left(0\right)},\lambda_{2}^{\left(0\right)},\ldots ,\lambda_{N}^{\left(0\right)}\right]}^{T}, & \lambda^{\left(1\right)}={\left[\lambda_{1}^{\left(1\right)},\lambda_{2}^{\left(1\right)},\ldots ,\lambda_{k}^{\left(1\right)}\right]}^{T}, & \lambda^{\left(2\right)}={\left[\lambda_{1}^{\left(1\right)},\lambda_{2}^{\left(1\right)},\ldots ,\lambda_{K}^{\left(1\right)}\right]}^{T}\\ \lambda^{\left(3\right)}=[\lambda_{1}^{\left(2\right)},\lambda_{2}^{\left(2\right)},\ldots ,\lambda_{L}^{\left(2\right)}], & \lambda^{\left(4\right)}={\left[\lambda_{1}^{\left(3\right)},\lambda_{2}^{\left(3\right)},\ldots ,\lambda_{K}^{\left(3\right)}\right]}^{T} & \end{array}$$

The Equation (15) can be decomposed into two layers of sub-problems. In the lower layer, we can get *K* sub-problems:

(17) $$\begin{array}{l} \Phi^{k}(\lambda^{\left(0\right)},\lambda^{\left(1\right)},\lambda^{\left(2\right)},\lambda^{\left(3\right)},\lambda^{\left(4\right)})=\underset{\text{P},\text{ρ}}{\max}\overset{N}{\underset{n=1}{\text{∑}}}L_{k}^{n,\text{SP}1(n),\text{SP}2(n)}\rho_{kn}\\ \text{subject to}\;\rho_{kn}\in \{0,1\},\forall n\in \{1,2,\ldots ,N\},\forall k\in \{1,2,\ldots K\}\\ \text{ρ}=\{\rho_{kn}\},\text{P}=\{P_{k1,1}^{n,\text{SP}1(n)},P_{k1,2}^{n,\text{SP}2(n)},P_{k2,1}^{n,\text{SP}1(n)},P_{k2,2}^{n,\text{SP}2(n)}\}\geq 0\\ \text{where}\\ L_{k}^{n,\text{SP}1(n),\text{SP}2(n)}(P_{k1}^{n,\text{SP}1(n)},P_{k2}^{n,\text{SP}2(n)})=\omega_{k}(I_{k1}^{n,\text{SP}1(n)}+I_{k2}^{n,\text{SP}2(n)})-\overset{L}{\underset{l=1}{\text{∑}}}\lambda_{l}^{\left(3\right)}[P_{k1,1}^{n,\text{SP}1(n)}S_{k1,pl}^{n}+P_{k2,2}^{n,\text{SP}2(n)}S_{k2,pl}^{n}\\ +P_{k1,2}^{n,\text{SP}2(n)}S_{k1,pl}^{\text{SP}2(n)}+P_{k2,1}^{n,\text{SP}1(n)}S_{k2,pl}^{\text{SP}1(n)}]-\lambda_{n}^{\left(0\right)}-\lambda_{n}^{\left(1\right)}P_{k1}^{n,\text{SP}1(n)}-\lambda_{k}^{\left(2\right)}P_{k2}^{n,\text{SP}2(n)}+\lambda_{k}^{\left(4\right)}(I_{k1}^{n,\text{SP}1(n)}+I_{k2}^{n,\text{SP}2(n)})\\ \forall n=1,2,\ldots ,N,\forall k=1,2,\cdots ,K \end{array}$$

We suppose that Γ*_k_* is the maximum value of the objective function in the lower layer. The master problem in the upper layer could be expressed as:

(18) $$\begin{array}{l} \text{H}(\text{P})=\underset{\underset{\lambda^{\left(2\right)},\lambda^{\left(3\right)},\lambda^{\left(4\right)}}{\lambda^{\left(0\right)},\lambda^{\left(1\right)}}}{\min}\overset{k}{\underset{k=1}{\text{∑}}}\Gamma_{k}+\overset{N}{\underset{n=1}{\text{∑}}}\lambda_{n}^{\left(0\right)}+\frac{1}{2}\overset{K}{\underset{k=1}{\text{∑}}}\lambda_{k}^{\left(1\right)}P_{t}+\frac{1}{2}\overset{K}{\underset{k=1}{\text{∑}}}\lambda_{k}^{\left(2\right)}P_{t}+\overset{L}{\underset{l=1}{\text{∑}}}\lambda_{l}^{\left(3\right)}Ith^{\left(l\right)}-\overset{K}{\underset{k=1}{\text{∑}}}\lambda_{k}^{\left(4\right)}R_{k}\\ \text{subject to}\;\{\lambda_{n}^{\left(0\right)},\lambda_{k}^{\left(1\right)},\lambda_{k}^{\left(2\right)},\lambda_{l}^{\left(3\right)},\lambda_{k}^{\left(4\right)}\}\geq 0 \end{array}$$

Since a dual function is always optimized by first optimizing some variables and then optimizing the remaining ones. We define a subcarrier pairing parameter *β_n,m_* ∈ {0,1} that takes 1 if the *n*th subcarrier in T1 is pairing to *m*th subcarrier in T2 and 0 otherwise. We first optimize the primal variables with the assumption that dual variables $\left\{\lambda_{n}^{\left(0\right)},\lambda_{k}^{\left(1\right)},\lambda_{k}^{\left(2\right)},\lambda_{l}^{\left(3\right)},\lambda_{k}^{\left(4\right)}\right\}$ are given. The resource allocation and subcarrier pairing process can be divided into four stages:

  (a) Allocating the optimal power factor $\left\{P_{k1,1}^{n,\text{SP}1(n)},P_{k1,2}^{n,\text{SP}2(n)},P_{k2,1}^{n,\text{SP}1(n)},P_{k2,2}^{n,\text{SP}2(n)}\right\}$ for SUs. $P_{k1,1}^{n,\text{SP}1(n)}$ and $P_{k2,2}^{n,\text{SP}2(n)}$ imply the power used for self-data transmission, respectively $P_{k1,2}^{n,\text{SP}2(n)}$ and $P_{k2,1}^{n,\text{SP}1(n)}$ imply the power used for partner-data transmission, respectively.
4. Allocating the optimal set of subcarriers Ω*_k_* for *k*th cooperation partner, *i.e.*, obtaining the optimal subcarrier allocation factor *ρ_kn_*.
5. Optimal pairing process for the subcarriers which are allocated to Ω*_k_*, *i.e.*, allocating the optimal subcarrier pairing factor *β_n,m_*.
6. After the temporary optimal primal variables have been obtained in each iteration process, we would find the temporary optimal dual variables $\left\{\lambda_{n}^{\left(0\right)},\lambda_{k}^{\left(1\right)},\lambda_{k}^{\left(2\right)},\lambda_{l}^{\left(3\right)},\lambda_{k}^{\left(4\right)}\right\}$, which can minimize the objective function H(**P**) as shown in Equation (18).

#### 3.1.1. Power Allocation Algorithm

Let $R_{k}^{n,\text{SP}1(n),\text{SP}2(n)}(P_{k1}^{n,\text{SP}1(n)},P_{k2}^{n,\text{SP}2(n)})=I_{k1}^{n,\text{SP}1(n)}+I_{k2}^{n,\text{SP}2(n)}$, if we make *ρ_kn_* = 1, the power allocation can be determined in a water-filling fashion. Taking derivatives of $L_{k}^{n,\text{SP}1(n),\text{SP}2(n)}$ with respect to $P_{k1}^{n,\text{SP}1(n)}$, $P_{k2}^{n,\text{SP}2(n)}$.

(19) $$\begin{array}{l} \frac{\partial L_{k}^{n,SP1(n),\text{SP}2(n)}(P_{k1}^{n,\text{SP}1(n)},P_{k2}^{n,\text{SP}2(n)})}{\partial P_{k1}^{n,\text{SP}1(n)}}=(\omega_{k}+\lambda_{k}^{\left(4\right)})\frac{\partial R_{k}^{n,\text{SP}1(n),SP2(n)}}{\partial P_{k1}^{n,\text{SP}1(n)}}-\lambda_{k}^{\left(4\right)}-\overset{L}{\underset{l=1}{\text{∑}}}\lambda_{l}^{\left(3\right)}[A_{k1,1}^{n,SP1(n)}S_{k1,pl}^{n}+F_{k2,1}^{n,SP1(n)}S_{k2,pl}^{n,SP1(n)}]=0\\ \frac{\partial L_{k}^{n,SP1(n),SP2(n)}(P_{k1}^{n,SP1(n)},P_{k2}^{n,\text{SP}2(n)})}{\partial P_{k1}^{n,SP1(n)}}=(\omega_{k}+\lambda_{k}^{\left(4\right)})\frac{\partial R_{k}^{n,SP1(n),SP2(n)}}{\partial P_{k2}^{n,SP2(n)}}-\lambda_{k}^{\left(2\right)}-\overset{L}{\underset{l=1}{\text{∑}}}\lambda_{l}^{\left(3\right)}[D_{k1,2}^{n,SP2(n)}S_{k1,pl}^{\text{SP}2(n)}+B_{k2,2}^{n,SP2(n)}S_{k2,pl}^{n}]=0\\ \text{where}\\ A_{k1,1}^{n,\text{SP}1(n)}=\frac{\gamma_{k2,0}^{\text{SP}1(n)}}{\gamma_{k1,k2}^{n}-\gamma_{k1,0}^{n}+\gamma_{k2,0}^{\text{SP}1(n)}},B_{k2,2}^{n,\text{SP}2(n)}=\frac{\gamma_{k1,0}^{\text{SP}2(n)}}{\gamma_{k2,k1}^{n}-\gamma_{k2,0}^{n}+\gamma_{k1,0}^{\text{SP}2(n)}}\\ D_{k1,2}^{n,\text{SP}2(n)}=\frac{\gamma_{k2,k1}^{n}-\gamma_{k2,0}^{n}}{\gamma_{k2,k1}^{n}-\gamma_{k2,0}^{n}+\gamma_{k1,0}^{\text{SP}2(n)}},F_{k2,1}^{n,\text{SP}1(n)}=\frac{\gamma_{k1,k2}^{n}-\gamma_{k1,0}^{n}}{\gamma_{k1,k2}^{n}-\gamma_{k1,0}^{n}+\gamma_{k2,0}^{\text{SP}1(n)}} \end{array}$$

Taking derivatives of $R_{k}^{n,\text{SP}1(n),\text{SP}2(n)}=I_{k1}^{n,\text{SP}1(n)}+I_{k2}^{n,\text{SP}2(n)}$ with respect to $P_{k1}^{n,\text{SP}1(n)}$, $P_{k2}^{n,\text{SP}2(n)}$, and according to Equation (19), we can get:

(20) $$\begin{array}{l} \frac{\partial R_{k}^{\text{n},\text{SP}1(\text{n}),SP2(\text{n})}}{\partial P_{k1}^{\text{n},SP1(\text{n})}}=\frac{\Delta f}{4\;\text{ln}\;2}\frac{\eta_{k1}^{\text{n},\text{SP}1(\text{n})}}{1+\eta_{k1}^{n,\text{SP}1(n)}P_{k1}^{n,\text{SP}1(n)}}=\frac{\Theta_{k,1}^{n,\text{SP}1(n)}}{\omega_{k}+\lambda_{k}^{\left(4\right)}}\\ \Theta_{k,1}^{n,\text{SP}1(n)}=\lambda_{k}^{\left(1\right)}+\overset{L}{\underset{l=1}{\text{∑}}}\lambda_{l}^{\left(3\right)}[A_{k1,1}^{n,\text{SP}1(n)}S_{k1,pl}^{n}+F_{k2,1}^{n,\text{SP}1(n)}S_{k2,pl}^{\text{SP}1(n)}]\\ \frac{\partial R_{k}^{n,SP1(n),SP2(n)}}{\partial P_{k2}^{n,SP2(n)}}=\frac{\Delta f}{4\;\text{ln}\;2}\frac{\eta_{k2}^{n,\text{SP}2(n)}}{1+\eta_{k2}^{n,\text{SP}2(n)}P_{k2}^{n,\text{SP}2(n)}}=\frac{\Theta_{k,2}^{n,\text{SP}2(n)}}{\omega_{k}+\lambda_{k}^{\left(4\right)}}\\ \Theta_{k,2}^{n,\text{SP}2(n)}=\lambda_{k}^{\left(2\right)}+\overset{L}{\underset{l=1}{\text{∑}}}\lambda_{l}^{\left(3\right)}[D_{k1,2}^{n,\text{SP}2(n)}S_{k1,pl}^{\text{SP}2(n)}+B_{k2,2}^{n,\text{SP}2(n)}S_{k2,pl}^{n}] \end{array}(20)$$

Together with the constraint $P_{1}^{n,SP1(n)}$, $P_{2}^{n,SP2(n)}\geq 0$, the temporary optimal solution can be obtained:

(21) $$\begin{array}{l} \underline{P_{k1}^{\text{n},\text{SP}1(n)}}=\max\left\{0,\frac{\frac{\Delta f}{4\;\ln\;2}\eta_{k1}^{n,\text{SP}1(n)}(\omega_{k}+\lambda_{k}^{\left(4\right)})-\Theta_{k,1}^{n,\text{SP}1(n)}}{\Theta_{k,1}^{n,\text{SP}1(n)}\eta_{k1}^{n,\text{SP}1(n)}}\right\}\\ \underline{P_{k2}^{n,\text{SP}2(n)}}=\max\left\{0,\frac{\frac{\Delta f}{4\;\ln\;2}\eta_{k2}^{n,\text{SP}2(n)}(\omega_{k}+\lambda_{k}^{\left(4\right)})-\Theta_{k,2}^{n,\text{SP}2(n)}}{\Theta_{k,2}^{n,\text{SP}2(n)}\eta_{k2}^{n,\text{SP}2(n)}}\right\} \end{array}$$

The temporary SU's transmit power can be obtained for the given dual variables:

(22) $$\begin{array}{ll} \underline{P_{k1,1}^{n,\text{SP}1(n)}}=A_{k1,1}^{n,\text{SP}1(n)}\underline{P_{k1}^{n,\text{SP}1(n)}}, & \underline{P_{k1,1}^{n,\text{SP}1(n)}}=F_{k2,1}^{n,\text{SP}1(n)}\underline{P_{k1}^{n,\text{SP}1(n)}}\\ \underline{P_{k1,2}^{n,\text{SP}2(n)}}=D_{k1,2}^{n,\text{SP}2(n)}\underline{P_{k2}^{n,\text{SP}2(n)}}, & \underline{P_{k2,2}^{n,\text{SP}2(n)}}=B_{k2,2}^{n,\text{SP}2(n)}\underline{P_{k2}^{n,\text{SP}2(n)}} \end{array}$$

#### 3.1.2. Subcarrier Allocation Algorithm

The subcarrier allocation constraint is that each subcarrier is allocated to no more than one SU cooperative partner, which prevents mutual interference among SUs. According to Section 3.1.1, we can get a temporary optimum power $\left\{\underline{P_{k1,1}^{n,\text{SP}1(n)}},\underline{P_{k2,1}^{n,\text{SP}1(n)}},\underline{P_{k1,2}^{n,\text{SP}2(n)}},\underline{P_{k2,2}^{n,\text{SP}2(n)}}\right\}$. We substitute this temporary optimum power vector into the objective function $L_{k}^{n,\text{SP}1(n),\text{SP}2(n)}(P_{k1}^{n,\text{SP}1(n)},P_{k2}^{n,\text{SP}2(n)})$ and the objective function $R_{k}^{n,\text{SP}1(n),\text{SP}2(n)}(P_{k1}^{n,\text{SP}1(n)},P_{k2}^{n,\text{SP}2(n)})$ to obtain the temporary max value $\underline{L_{k}^{n,\text{SP}1(n),\text{SP}2(n)}}$ and $\underline{R_{k}^{n,\text{SP}1(n),\text{SP}2(n)}}$, respectively. Taking account of resource fairness, we can formulate the optimization problem of subcarrier allocation as:

(23) $$\begin{array}{l} \underset{\text{ρ}}{\max}h(\text{ρ})=\overset{K}{\underset{k=1}{\text{∑}}}\overset{N}{\underset{n=1}{\text{∑}}}\rho_{kn}\underline{L_{k}^{n,\text{SP}1(n),\text{SP}2(n)}}+\frac{\zeta {\left\{\overset{K}{\underset{k=1}{\text{∑}}}\overset{N}{\underset{n=1}{\text{∑}}}\rho_{kn}\underline{R_{k}^{n,\text{SP}1(n),\text{SP}2(n)}}\right\}}^{2}}{K\overset{K}{\underset{k=1}{\text{∑}}}{\left(\overset{N}{\underset{n=1}{\text{∑}}}\rho_{kn}\underline{R_{k}^{n,\text{SP}1(n),\text{SP}2(n)}}\right)}^{2}}\\ \text{subject to}\;\rho_{kn}\in \{0,1\},\overset{k}{\underset{k=1}{\text{∑}}}\rho_{kn}\leq 1,\forall k,n,\\ \text{where}\;\text{ρ}=\{\rho_{kn}\},1\leq k\leq K,1\leq n\leq N \end{array}$$

where the value *ζ* is the weighting factor to balance the total transmitted data rate and fairness index among SUs. The bigger the value *ζ* is, the greater the fairness can be obtained, and otherwise the greater the transmitted data rate is. It is a mixed binary integer programming problem that is difficult to solve. To reduce the exponential computational complexity, a suboptimum subcarrier allocation algorithm with less computational complexity is developed. The pseudo-code of subcarrier allocation algorithm can be described as follows:

(1) Initialization
  (i) Make *ρ_kn_* = 0, Ω*_k_* = Ø, ∀*k*, *n*
(2) For *n* = 1 to *N*
  (i) For *k* = 1 to *K*
    a) Make *ρ_kn_* = 1
    b) Assign *V_nk_* = *h*(**ρ**) according to Equation (23)
    c) Make *ρ_kn_* = 0.
  (ii) Assign $k^{*}=\underset{k}{\arg\max}V_{nk}$
  (iii) Assign ρ_k*n_ = 1, Ω_k__*_ = Ω_k__*_ ∪ {*n*}, *ρ_kn_*= 0, ∀*k* ≠ k*

According to Section 3.1.2, we can obtain a temporary subcarrier allocation vector $\underline{\text{ρ}}=\{\underline{\rho_{kn}},1\leq k\leq K,1\leq n\leq N\}$ for the given dual variables.

#### 3.1.3. Subcarrier Pairing Algorithm

The pairing constraint is that each subcarrier m in listening phase only pairs with at most one subcarrier *n* in the relaying phase. We assume that the pairing for deferent frames is not the same. The pairing process of the subcarrier allocated to *k*th cooperation partner can be expressed as:

(24) $$\begin{array}{l} \text{Frame}1:\max\sum_{n\in \Omega_{k}}\sum_{m\in \Omega_{k}}\beta_{n,m}\;I_{k1}^{n,m};\;\text{Frame}2:\max\sum_{n\in \Omega_{k}}\sum_{m'\in \Omega_{k}}\beta_{n,m'}\;I_{k2}^{n,m'};\\ \text{subject to}\;\text{C}6:\sum_{m\in \Omega_{k}}\beta_{n,m}\leq 1,\text{C}7:\sum_{m'\in \Omega_{k}}\beta_{n,m'}\leq 1,\forall n\in \Omega_{k}\\ \text{C8}:\sum_{n\in \Omega_{k}}\beta_{n,m}\leq 1,\text{C9}:\sum_{n\in \Omega_{k}}\beta_{n,m'}\leq 1,\forall m\in \Omega_{k},\forall m'\in \Omega_{k}\\ \text{ C}10:\beta_{n,m}\in \{0,1\},\text{C}11:\beta_{n,m'}\in \{0,1\} \end{array}$$

Constraints **C**6, C8 and **C**10 correspond to the pairing constraint that each subcarrier *n* in listening phase only pairs with one subcarrier *m* in the relaying phase in the first frame. Constraint **C**7, C9 and **C**11 correspond to the pairing constraint that each subcarrier *n* in listening phase only pairs with one subcarrier *m'* in the relaying phase for the second frame. We can obtain the temporary optimal *m*, *m′* for any *n* as:

(25) $$\underline{m}=\underset{m}{\arg\max}\{I_{k1}^{n,m}\},\underline{m\prime }=\underset{m\prime }{\arg\max}\{I_{k2}^{n,m\prime }\}$$

That is:

(26) $$\begin{array}{l} \text{Frame}1:\beta_{n,\underline{m}}=1,\text{SP}1(n)=\underline{m},\beta_{n,m}=0\forall m\neq \underline{m}\\ \text{Frame}2:\beta_{n,\underline{m'}}=1,\text{SP}2(n)=\underline{m'},\beta_{n,m'}=0\forall m'\neq \underline{m'} \end{array}$$

The subcarrier pairing scheme can be shown as following:

For *k* = 1 to *K*

(a) Δ = Ω*_k_*, Δ′ = Ω*_k_*, *β_n,m_* = 0, *β_n,m_*_′_ = 0, ∀ *n*,*m*,*m*′ ∈ Ω*_k_*
(b) While Δ ≠ Ø, Δ′ ≠ Ø
  (i) *n* ∈ Δ, *n*′ ∈ Δ′
  (ii) Find *m* ∈ Δ, *m*′ ∈ Δ′ satisfying $I_{k1}^{n,m}\geq I_{k1}^{n,l}$, $I_{k2}^{n,m'}\geq I_{k2}^{n,l'}$, ∀*l* ∈ Δ, ∀*l'* ∈ Δ′
  (iii) Assign
    SP1 (*n*) = *m, β_n_*_,_*_m_* = 1, Δ = Δ − {*n*} − {*m*}
    SP1 (*n*′) = *m*′, *β_n_*_′_,*_m_*_′_ = 1, Δ′ = Δ′ − {*n*′ } − {*m*′ }

Through Section 3.1.3, we can obtain a temporary optimal subcarrier pairing vector $\underline{\beta_{n,m}}$, $\underline{\beta_{n,m^{\prime }}}$, 1 ≤ {*n*, *m*, *m*′ } ≤ *N* for the given dual variables.

#### 3.1.4. Optimizing the Dual Variables

The optimal values of dual variables can be achieved iteratively by the sub-gradient method as follows:

(27) $$\begin{array}{l} \lambda_{n}^{(0),i+1}={\left\{\lambda_{n}^{(0),i}-\alpha_{0,n}^{\left(i\right)}[1-\overset{k}{\underset{k=1}{\text{∑}}}\rho_{kn}]\right\}}^{+}\\ \lambda_{k}^{(1),i+1}={\left\{\lambda_{k}^{(1),i}-\alpha_{1,k}^{\left(i\right)}[\frac{P_{t}}{2}-\overset{N}{\underset{n=1}{\text{∑}}}\rho_{kn}P_{k1}^{n,\text{SP}1(n)}]\right\}}^{+}\\ \lambda_{k}^{(2),i+1}={\left\{\lambda_{k}^{(2),i}-\alpha_{2,k}^{\left(i\right)}[\frac{P_{t}}{2}-\overset{N}{\underset{n=1}{\text{∑}}}\rho_{kn}P_{k2}^{n,\text{SP}2(n)}]\right\}}^{+}\\ \lambda_{l}^{(3),i+1}={\left\{\lambda_{l}^{(3),i}-\alpha_{3,l}^{\left(i\right)}[Ith^{\left(l\right)}-\overset{K}{\underset{k=1}{\text{∑}}}\overset{N}{\underset{n=1}{\text{∑}}}\rho_{kn}(P_{k1,1}^{n,\text{SP}1(n)}S_{k1,pl}^{n}+P_{k2,2}^{n,\text{SP}2(n)}S_{k2,pl}^{n}+P_{k1,2}^{n,\text{SP}2(n)}S_{k1,pl}^{\text{SP}2(n)}+P_{k2,1}^{n,\text{SP}1(n)}S_{k2,pl}^{\text{SP}1(n)}]\right\}}^{+}\\ \lambda_{k}^{(4),i+1}={\left\{\lambda_{k}^{(4),i}-\alpha_{4,k}^{\left(i\right)}[\overset{N}{\underset{n=1}{\text{∑}}}\rho_{kn}(I_{k1}^{n,\text{SP}1(n)}+I_{k2}^{n,\text{SP}2(n)})-R_{k}]\right\}}^{+} \end{array}$$

where $\left\{\alpha_{0,n}^{\left(i\right)},\alpha_{1,k}^{\left(i\right)},\alpha_{2,k}^{\left(i\right)},\alpha_{3,l}^{\left(i\right)},\alpha_{4,k}^{\left(i\right)}\right\}$ is a small positive step size for the *i*th iteration, *i* is the iteration number and ${\left(x\right)}^{+}\overset{\Delta }{=}\max(x,0)$, with the appropriate step sizes, the iterations are convergent. The remaining issue is how to determine the step size $\left\{\alpha_{0,n}^{\left(i\right)},\alpha_{1,k}^{\left(i\right)},\alpha_{2,k}^{\left(i\right)},\alpha_{3,l}^{\left(i\right)},\alpha_{4,k}^{\left(i\right)}\right\}$. Clearly, performing a line search at each iteration process perform well. For a given current iteration ${\text{λ}}^{i}=\{\lambda_{n}^{(0),i},\lambda_{k}^{(1),i},\lambda_{k}^{(2),i},\lambda_{l}^{(3),i},\lambda_{k}^{(4),i}\}$ and a search direction **d**^(*i*)^, we compute step size **α**^*k*,(*i*)^ by:

(28) $$\begin{array}{l} \{\underline{\alpha_{0,n}^{\left(i\right)}},\underline{\alpha_{1,k}^{\left(i\right)}},\underline{\alpha_{2,k}^{\left(i\right)}},\underline{\alpha_{3,l}^{\left(i\right)}},\underline{\alpha_{4,k}^{\left(i\right)}}\}=\underset{\alpha_{0,n}^{\left(i\right)},\alpha_{1,k}^{\left(i\right)},\alpha_{2,k}^{\left(i\right)},\alpha_{3,l}^{\left(i\right)},\alpha_{4,k}^{\left(i\right)}}{\arg\min}\text{Q}\{\lambda_{n}^{\left(0,i\right)}+\alpha_{0,n}^{\left(i\right)}d_{0}^{\left(i\right)}\\ ,\lambda_{k}^{(1),i}+\alpha_{1,k}^{\left(i\right)}d_{1}^{\left(i\right)},\lambda_{k}^{(2),i}+\alpha_{2,k}^{\left(i\right)}d_{2}^{\left(i\right)},\lambda_{l}^{(3),i}+\alpha_{3,k}^{\left(i\right)}d_{3}^{\left(i\right)},\lambda_{k}^{(4),i}+\alpha_{4,k}^{\left(i\right)}d_{4}^{\left(i\right)}\}\\ \text{where}\;Q(\lambda_{n}^{\left(0\right)},\lambda_{k}^{\left(1\right)},\lambda_{k}^{\left(2\right)},\lambda_{l}^{\left(3\right)},\lambda_{k}^{\left(4\right)}=\overset{K}{\underset{k=1}{\text{∑}}}\Gamma_{k}+\overset{N}{\underset{n=1}{\text{∑}}}\lambda_{n}^{\left(0\right)}+\overset{K}{\underset{k=1}{\text{∑}}}\lambda_{k}^{\left(1\right)}P_{t}\\ +\overset{K}{\underset{k=1}{\text{∑}}}\lambda_{k}^{\left(2\right)}P_{t}+\overset{L}{\underset{l=1}{\text{∑}}}\lambda_{l}^{\left(3\right)}Ith^{\left(l\right)}-\overset{K}{\underset{k=1}{\text{∑}}}\lambda_{k}^{\left(4\right)}R_{k},\forall k,n \end{array}$$

Within each iteration process, the power allocation vectors can be updated respectively by Equation (22), the subcarrier allocation vectors can be updated respectively by subcarrier allocation algorithm shown in Section 3.1.2, the subcarrier pairing vectors can be updated respectively by subcarrier pairing algorithm which is shown in Section 3.1.3, with the updated value $\left\{\lambda_{n}^{(0),i},\lambda_{k}^{(1),i},\lambda_{k}^{(2),i},\lambda_{l}^{(3),i},\lambda_{k}^{(4),i}\right\}$. Therefore, the dual variable **λ***^i^* will converge to the dual optimum *λ̳* as *i* → ∞ and the temporary primal optimum variable will also converge to the primal optimum value after several iterations, *i.e.*, temporary vector $\left\{\underline{P_{k1,1}^{n,\text{SP}1(n)}},\underline{P_{k2,1}^{n,\text{SP}1(n)}},\underline{P_{k1,2}^{n,\text{SP}2(n)}},\underline{P_{k2,2}^{n,\text{SP}2(n)}}\right\}$ will converge to optimal vector $\left\{\underset{̳}{P_{k1,1}^{n,\text{SP}1(n)}},\underset{̳}{P_{k1,2}^{n,\text{SP}2(n)}},\underset{̳}{P_{k2,1}^{n,\text{SP}1(n)}},\underset{̳}{P_{k2,2}^{n,\text{SP}2(n)}}\right\}$, temporary optimal vector $\underline{\text{ρ}}=\{\underset{̳}{\rho_{kn}},1\leq k\leq K,1\leq n\leq N\}$ will converge to the optimal vector $\underset{̳}{\text{ρ}}=\{\underset{̳}{\rho_{kn}},1\leq k\leq K,1\leq n\leq N\}$, temporary vector $\underline{\beta_{n,m}}$, $\underline{\beta_{n,m^{\prime }}}$ will converge to the optimal vector $\underset{̳}{\beta_{n,m}}$, $\underset{̳}{\beta_{n,m^{\prime }}}$.

### 3.2. Resource Allocation with Partial CSI

If full CSI can be achieved at the *k*1th and *k*2th (1 ≤ *k* ≤ *K*) SU transmitter, the optimal subcarrier allocation vector **ρ̳**, subcarrier pairing vector $\underset{̳}{\beta_{n,m}}$, $\underset{̳}{\beta_{n,m^{\prime }}}$, the power allocation vector $\left\{\underset{̳}{P_{k1,1}^{n,\text{SP}1(n)}},\underset{̳}{P_{k1,2}^{n,\text{SP}2(n)}},\underset{̳}{P_{k2,1}^{n,\text{SP}1(n)}},\underset{̳}{P_{k2,2}^{n,\text{SP}2(n)}}\right\}$ can be determined simply by the proposed algorithm which is shown in Section 3.1. However, the practical case in which only partial CSI of the wireless channel between the secondary base station and SUs is available have to be considered. The CSI of non-adjacent link may be undesirable and even unavailable when the SUs are mobile. In this section, we investigate the optimal resource allocation in SU cooperation network with partial CSI at each transmitter. Specifically, the *ki*th SU transmitter has full CSI of its adjacent links $h_{ki,kj}^{ps,n}(i\neq j)$, $h_{ki,0}^{ps,n}$ but only statistical CSI of non-adjacent link $h_{ki,0}^{ps,n}$. We assume that the SUs can know $h_{pl,ki}^{ps,n}$, $h_{pl,0}^{ps,n}$, *i.e.*, $h_{pl,kj}^{ps,n},h_{pl,0}^{ps,n}$ are still available at SU transmitter, and the link between cooperative users is symmetric for simplicity, *i.e.*, $h_{ki,kj}^{ps,n}=h_{kj,ki}^{ps,n}$.

Under these assumptions and according to [47], the objective function:

(29) $$\Psi =\overset{K}{\underset{k=1}{\text{∑}}}\overset{N}{\underset{n=1}{\text{∑}}}\omega_{k}\rho_{kn}\{I_{k1}^{n,\text{SP}1(n)}(P_{k1}^{n,\text{SP}1(n)})+I_{k2}^{n,\text{SP}2(n)}(P_{k2}^{n,\text{SP}2(n)})\}$$

can be rewritten as:

(30) $$\Psi =\overset{K}{\underset{k=1}{\text{∑}}}\overset{N}{\underset{n=1}{\text{∑}}}\omega_{k}\rho_{kn}\{\ln\bar{I_{k1}^{n,\text{SP}1(n)}(P_{k1}^{n,\text{SP}1(n)})}+\ln\bar{I_{k2}^{n,\text{SP}2(n)}(P_{k2}^{n,\text{SP}2(n)})}\}$$

where $\bar{I_{k1}^{\text{SP}1(n)}}$ and $\bar{I_{k2}^{\text{SP}2(n)}}$ are defined as:

(31) $$\begin{array}{l} \bar{I_{k1}^{n,\text{SP}1(n)}(P_{1}^{n,\text{SP}1(n)})}=E_{h_{k2,0}^{ss,n}}[I_{k1}^{n,\text{SP}1(n)}(P_{k1}^{n,\text{SP}1(n)})]\\ \bar{I_{k2}^{n,\text{SP}2(n)}(P_{2}^{n,\text{SP}2(n)})}=E_{h_{k1,0}^{ss,n}}[I_{k2}^{n,\text{SP}2(n)}(P_{k2}^{n,\text{SP}2(n)})] \end{array}$$

In order to seek the optimal power allocation solution, we derive the explicit expressions for $\bar{I_{k1}^{n,\text{SP}1(n)}}$ and $\bar{I_{k2}^{n,\text{SP}2(n)}}$ described as:

Let $h_{ki,0}^{ss,n}={\left(\frac{1}{d_{ki,0}}\right)}^{v}x_{ki,0}$, *i*=1,2, where *d*_*ki*,0_ denotes the distance between the *k*ith SU and the AP, *v* is the path-loss exponent, *x*_*ki*,0_, (*i* = 1,2) is the normalized complex Gaussian random variable distributed as *CN* (0, 1), then at high signal-to-noise ratio (SNR):

(32) $$\begin{array}{l} \bar{I_{k1}^{n,\text{SP}1(n)}}=\frac{\Delta f}{4\;\ln\;2}\{\frac{P_{k1}^{n,\text{SP}1(n)}\gamma_{k1,k2}^{n}W_{1}+W_{1}}{\gamma_{k1,k2}^{n}-\gamma_{k1,0}^{n}}(1-\frac{P_{k1}^{n,\text{SP}1(n)}\gamma_{k1,k2}^{n}W_{1}+W_{1}}{\gamma_{k1,k2}^{n}-\gamma_{k1,0}^{n}})-\frac{W_{1}}{\gamma_{k1,k2}^{n}-\gamma_{k1,0}^{n}}(1-\frac{W_{1}}{\gamma_{k1,k2}^{n}-\gamma_{k1,0}^{n}})\}\\ \bar{I_{k2}^{n,\text{SP}2(n)}}=\frac{\Delta f}{4\;\ln\;2}\{\frac{P_{k2}^{n,\text{SP}2(n)}\gamma_{k2,k1}^{n}W_{2}+W_{2}}{\gamma_{k2,k1}^{n}-\gamma_{k2,0}^{n}}(1-\frac{P_{k2}^{n,\text{SP}2(n)}\gamma_{k2,k1}^{n}W_{2}+W_{2}}{\gamma_{k2,k1}^{n}-\gamma_{k2,0}^{n}})-\frac{W_{2}}{\gamma_{k2,k1}^{n}-\gamma_{k2,0}^{n}}(1-\frac{W_{1}}{\gamma_{k2,k1}^{n}-\gamma_{k2,0}^{n}})\} \end{array}$$

where *W*_1_,*W*_2_ in Equation (32) are defined as:

(33) $$W_{1}={\left[(\sigma^{2}+\overset{L}{\underset{l=1}{\text{∑}}}J_{pl,0}^{\text{SP}1(n)})d_{k2,0}^{2v}\right]}^{-1},w_{2}={\left[(\sigma^{2}+\overset{L}{\underset{l=1}{\text{∑}}}J_{pl,0}^{\text{SP}1(n)})d_{k1,0}^{2v}\right]}^{-1}$$

Proof: It is sufficient to show $\bar{I_{k1}^{n,\text{SP}1(n)}}$, and $\bar{I_{k2}^{n,\text{SP}2(n)}}$ can be derived in exactly the same way, then

(34) $$\begin{array}{l} \bar{I_{k1}^{n,\text{SP}1(n)}}=E_{h_{k2,0}^{ss,n}}[I_{k1}^{\text{SP}1(n)}]=E_{h_{k2,0}^{ss,n}}\{\frac{\Delta f}{4}\log_{2}(1+\eta_{k1}^{n,\text{SP}1(n)}P_{k1}^{n,\text{SP}1(n)})\}\\ =E_{h_{k2,0}^{ss,n}}\{\frac{\Delta f}{4}\log_{2}(1+\frac{\gamma_{k1,k2}^{n}\gamma_{k2,0}^{\text{SP}1(n)}}{\gamma_{k1,k2}^{n}-\gamma_{k1,0}^{n}+\gamma_{k2,0}^{\text{SP}1(n)}}P_{k1}^{n,\text{SP}1(n)})\}\\ =E_{h_{k2,0}^{ss,n}}\left\{\frac{\Delta f}{4}\log_{2}(1+\frac{\gamma_{k1,k2}^{n}\frac{{\left|h_{k2,0}^{ss,\text{SP}1(n)}\right|}^{2}}{\sigma^{2+}\overset{L}{\underset{l=1}{\text{∑}}}J_{pl,0}^{\text{SP}1(n)}}}{\gamma_{k1,k2}^{n}-\gamma_{k1,0}^{n}+\frac{{\left|h_{k2,0}^{ss,\text{SP}1(n)}\right|}^{2}}{\sigma^{2+}\overset{L}{\underset{l=1}{\text{∑}}}J_{pl,0}^{\text{SP}1(n)}}}P_{k1}^{n,\text{SP}1(n)})\right\}\\ =\frac{\Delta f}{4}E_{h_{k2,0}^{ss,n}}\left\{\log_{2}(1+\frac{\gamma_{k1,k2}^{n}[{\left(\sigma^{2}+\overset{L}{\underset{l=1}{\text{∑}}}J_{pl,0}^{n})d_{k2,0}^{2v}\right]}^{-1}{\left|x_{k2,0}\right|}^{2}P_{k1}^{n,\text{SP}1(n)})}{\left(\gamma_{k1,k2}^{n}-\gamma_{k1,0}^{n}+\frac{{\left|x_{k2,0}\right|}^{2}}{(\sigma^{2+}\overset{L}{\underset{l=1}{\text{∑}}}J_{pl,0}^{\text{SP}1(n)})d_{k2,0}^{2v}}\right)}\right\} \end{array}$$

The third equality holds since we assume each transmission block is long enough to undergo different channel realizations as argued in [48]. Next, let:

(35) $$W_{1}={\left[(\sigma^{2}+\overset{L}{\underset{l=1}{\text{∑}}}J_{pl,0}^{\text{SP}1(n)})d_{k2,0}^{2v}\right]}^{-1},y={\left|x_{k2,0}\right|}^{2}$$

then according to [49], we can obtain:

(36) $$\begin{array}{l} \bar{I_{k1}^{n,\text{SP}1(n)}}=E_{h_{k2,0}^{ss,n}}[I_{k1}^{n,\text{SP}1(n)}]=\frac{\Delta f}{4}E_{h_{k2,0}^{ss,n}}\left\{\log_{2}[1+\frac{\gamma_{k1,k2}^{n}W_{1}yP_{k1}^{n,\text{SP}1(n)}}{\gamma_{k1,k2}^{n}-\gamma_{k1,0}^{n}+W_{1}y}]\right\}\\ =\frac{\Delta f}{4}\int_{0}^{+\infty }\log_{2}\left[1+\frac{\gamma_{k1,k2}^{n}W_{1}yP_{k1}^{n,\text{SP}1(n)}}{\gamma_{k1,k2}^{n}-\gamma_{k1,0}^{n}+W_{1}y}\right]e^{-y}dy\\ =\frac{\Delta f}{4\;\ln\;2}\int_{0}^{+\infty }\frac{e^{-y}P_{k1}^{n,\text{SP}1(n)}\gamma_{k1,k2}^{n}W_{1}(\gamma_{k1,k2}^{n}-\gamma_{k1,0}^{n})dy}{\left(\gamma_{k1,k2}^{n}-\gamma_{k1,0}^{n}+W_{1}y)(\gamma_{k1,k2}^{n}-\gamma_{k1,0}^{n}+W_{1}y+P_{k1}^{n,\text{SP}1(n)}\gamma_{k1,k2}^{n}W_{1}y\right)}\\ =\frac{\Delta f}{4\;\ln\;2}\int_{0}^{+\infty }\{\frac{e^{-y}}{y+\frac{\gamma_{k1,k2}^{n}-\gamma_{k1,0}^{n}}{W_{1}}}-\frac{e^{-y}}{y+\frac{\gamma_{k1,k2}^{n}-\gamma_{k1,0}^{n}}{P_{k1}^{n,\text{SP}1(n)}\gamma_{k1,k2}^{n}W_{1}+W_{1}}}\}dy\\ -=\frac{\Delta f}{4\;\ln\;2}\{-e^{\frac{\gamma_{k1,k2}^{n}-\gamma_{k1,0}^{n}}{W_{1}}}{\int_{-\infty }^{\frac{\gamma_{k1,0}^{n}-\gamma_{k1,k2}^{n}}{W_{1}}}\frac{e^{y}}{y}dy+e}^{\frac{\gamma_{k1,k2}^{n}-\gamma_{k1,0}^{n}}{P_{k1}^{n,\text{SP}1(n)}\gamma_{k1,k2}^{n}W_{1}+W_{1}}}\int_{-\infty }^{\frac{\gamma_{k1,0}^{n}-\gamma_{k1,k2}^{n}}{P_{k1}^{n,\text{SP}1(n)}\gamma_{k1,k2}^{n}W_{1}+W_{1}}}\frac{e^{y}}{y}dy\}\\ =\frac{-\Delta f}{4\;\ln\;2}\{-e^{\frac{\gamma_{k1,k2}^{n}-\gamma_{k1,0}^{n}}{W_{1}}}Ei(\frac{\gamma_{k1,0}^{n}-\gamma_{k1,k2}^{n}}{W_{1}})+e^{\frac{\gamma_{k1,k2}^{n}-\gamma_{k1,0}^{n}}{P_{k1}^{n,\text{SP}1(n)}\gamma_{k1,k2}^{n}W_{1}+W_{1}}}Ei(\frac{\gamma_{k1,0}^{n}-\gamma_{k1,k2}^{n}}{P_{k1}^{n,\text{SP}1(n)}\gamma_{k1,k2}^{n}W_{1}+W_{1}})\} \end{array}$$

where *Ei*(□) denotes the exponential integral function defined as $Ei(t)=\int_{-\infty }^{t}e^{x}x^{-1}dx$ and *E_i_*(*t*) can be expanded asymptotically as:

(37) $$Ei(t)\approx \frac{e^{t}}{t}(1-\overset{+\infty }{\underset{i=1}{\text{∑}}}\frac{\prod_{k=1}^{i}k}{x^{i}})\approx \frac{e^{t}}{t}[1+\frac{1}{t}+o(\frac{1}{t})]$$

Then using the assumption that SNR is high, we readily obtain Equation (32). This completes the proof.

We can use the resource allocation and subcarrier pairing algorithm which is proposed in Section 3.1 to solve the corresponding optimization Equation (14) once again. When partial CSI can be achieved by SUs, the optimal subcarrier allocation vector $\overset{︷}{\rho }$, subcarrier pairing vector $\overset{︷}{\beta_{n,m}}$, $\overset{︷}{\beta_{n,m'}}$ and optimal power allocation vector $\overset{︷}{P_{k1}^{n,\text{SP}1(n)}}$, $\overset{︷}{P_{k2}^{n,\text{SP}2(n)}}$ can be obtained. According to Equation (11), we can get

(38) $$\begin{array}{c} \overset{︷}{P_{k1,1}^{n,SP1(n)}}=E_{h_{k2,0}^{ss,n}}\left\{\frac{\gamma_{k2,0}^{SP1(n)}}{\gamma_{k1,k2}^{n}-\gamma_{k1,0}^{n}+\gamma_{k2,0}^{SP1(n)}}\overset{︷}{P_{k1}^{n,SP1(n)}}\right\}\\ =\int_{0}^{+\infty }\frac{W_{1}y}{\gamma_{k1,k2}^{n}-\gamma_{k1,0}^{n}+W_{1}y}\overset{︷}{P_{k1}^{n,SP1(n)}}e^{-y}dy\\ =\overset{︷}{P_{k1}^{n,SP1(n)}}\int_{0}^{+\infty }\left\{1-\frac{\gamma_{k1,k2}^{n}-\gamma_{k1,0}^{n}}{\gamma_{k1,k2}^{n}-\gamma_{k1,0}^{n}+W_{1}y}\right\}e^{-y}dy\\ =\overset{︷}{P_{k1}^{n,SP1(n)}}\left\{1+\frac{\left(\gamma_{k1,k2}^{n}-\gamma_{k1,0}^{n}\right)}{W_{1}}e\frac{\left(\gamma_{k1,k2}^{n}-\gamma_{k1,0}^{n}\right)}{W_{1}}\right\}\int_{-\infty }^{\frac{\gamma_{k1,0}^{n}-\gamma_{k1,k2}^{n}}{W_{1}}}\frac{e^{t}}{t}dt\\ =\overset{︷}{P_{k1}^{n,SP1(n)}}+\frac{\overset{︷}{P_{k1}^{n,SP1(n)}}(\gamma_{k1,k2}^{n}-\gamma_{k1,0}^{n})}{W_{1}}e\frac{\left(\gamma_{k1,k2}^{n}-\gamma_{k1,0}^{n}\right)}{W_{1}}Ei\left(\frac{\left(\gamma_{k1,0}^{n}-\gamma_{k1,k2}^{n}\right)}{W_{1}}\right)\\ =\frac{W_{1}}{\gamma_{k1,k2}^{n}-\gamma_{k1,0}^{n}}\overset{︷}{P_{k1}^{n,SP1(n)}} \end{array}$$

Then we also can get:

(39) $$\begin{array}{l} \overset{︷}{P_{k2,1}^{n,SP1(n)}}=\left(1-\frac{W_{1}}{\gamma_{k1,k2}^{n}-\gamma_{k1,0}^{n}}\right)\overset{︷}{P_{k1}^{n,SP1(n)}},\overset{︷}{P_{k1,2}^{n,SP2(n)}}\\ =\left(1-\frac{W_{2}}{\gamma_{k2,k1}^{n}-\gamma_{k2,0}^{n}}\right)\overset{︷}{P_{k2}^{n,SP2(n)}}\overset{︷}{P_{k2,2}^{n,SP2(n)}}=\frac{W_{2}}{\gamma_{k2,k1}^{n}-\gamma_{k2,0}^{n}}\overset{︷}{P_{k2}^{n,SP2(n)}} \end{array}$$

Intuitively, the system performance would be degraded due to limited CSI though this scheme which does not require each cooperative node to have full CSI of nonadjacent link, as verified in the numerical simulation.

### 3.3. Comparison with Classical Resource Allocation Algorithms

Several existing schemes are compared with the proposed subcarrier-pair based resource allocation algorithm in terms of the system transmitted rate and fairness, respectively. These existing schemes include the following:

LWF-PI-without-SP: The subcarrier is allocated according to the channel gain. The messages transmitted on subcarrier m at the source node will be retransmitted on the same subcarrier m at the relay node. The power is allocated according to LWF-PI algorithm [23] on each subcarrier.
EPA-without-SP: The subcarrier is allocated according to the channel gain. The messages transmitted on subcarrier m at the source node will be retransmitted on the same subcarrier m at the relay node. The power is allocated equally on each subcarrier.
LWF-PI-with-SP: The subcarrier is allocated according to the channel gain. The messages transmitted on subcarrier m at the source node will be retransmitted on subcarrier n, which is selected by proposed subcarrier pairing algorithm, at the relay node. The power is allocated according to LWF-PI algorithm [23] on each subcarrier.
EPA-with-SP: The subcarrier is allocated according to the channel gain. The messages transmitted on subcarrier m at the source node will be retransmitted on subcarrier n, which is selected by proposed subcarrier pairing algorithm at the relay node. The power is allocated equally on each subcarrier.
Optimal-Scheme-with-SP: The subcarrier is allocated according to the channel gain. The messages transmitted on subcarrier m at the source node will be retransmitted on subcarrier n, which is selected by proposed subcarrier pairing algorithm at the relay node. The power is allocated according to Optimal Scheme [37] on each subcarrier.
Partial CSI: the difference between the previous five algorithms and Partial CSI scheme is that the SU has only imperfect CSI of non-adjacent link under partial CSI scheme. In this case, the objective function is formulated by statistical methods.

## 4. Simulation Results

We have studied asymmetric or linear network with all SUs of *k*th cooperative partner located in the same line. Specifically, *k*1th SU and the destination AP are fixed at (0, 0), (1, 0) respectively, and *k*2th SU is located at (*d*, 0), 0 ≤ *d* ≤ 1, without loss of generality. The results for path loss exponent *ν* = 2 are presented and all channels are modeled as Rayleigh flat fading with AWGN. Some simulation parameters are shown in Table 3.

Simulation results are presented in this section to verify the performance of the proposed subcarrier-pair based resource allocation algorithm. In our simulations, the CVX, a package for specifying and solving convex programs, is used to solve formulated optimization resource allocation problems.

The channel gains $h_{ki,kj}^{ss,n}$, $h_{ki,0}^{ss,n}$, $h_{ki,pl}^{sp,n}$, $h_{pl,ki}^{ss,n}$, $h_{pl,0}^{ps,n}$ used in this paper are assumed to be Rayleigh fading, since the channel fading gains for different realizations of channel gain can be different, an average transmission capacity of 10,000 independent simulation runs is considered. And individual fairness index is defined as [46]:

(40) $$\text{fair}=\frac{{\left\{\overset{K}{\underset{k=1}{\text{∑}}}\overset{N}{\underset{n=1}{\text{∑}}}\rho_{kn}\underset{̳}{R_{k}^{n,\text{SP}1(n),\text{SP}2(n)}}\right\}}^{2}}{K{\left(\overset{K}{\underset{k=1}{\text{∑}}}\overset{N}{\underset{n=1}{\text{∑}}}\rho_{kn}\underset{̳}{R_{k}^{n,\text{SP}1(n),\text{SP}2(n)}}\right)}^{2}}$$

The fairness ranges between 0 and 1. The higher the value fairness is, the more fair the throughput distribution among SUs is.

## 4.1. The System Transmitted Data Rate Obtained under Resource Allocation Algorithms

**Figure 4.**
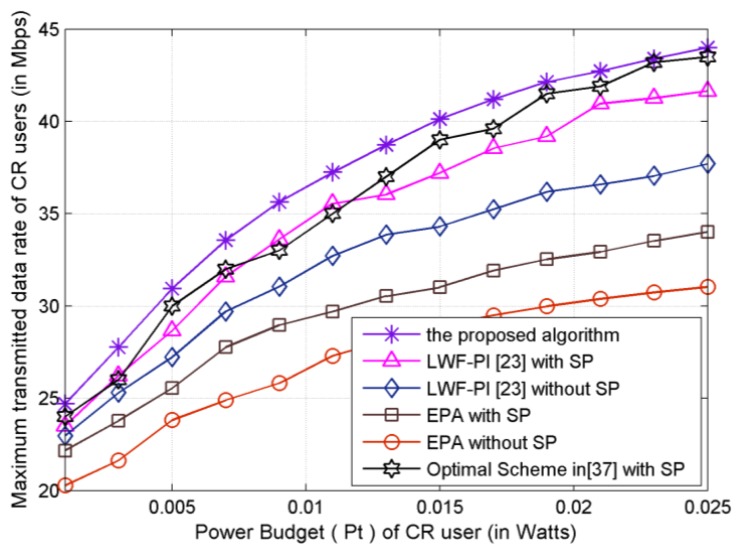
Maximum transmitted data rate *versus* Power Budget.

**Figure 5.**
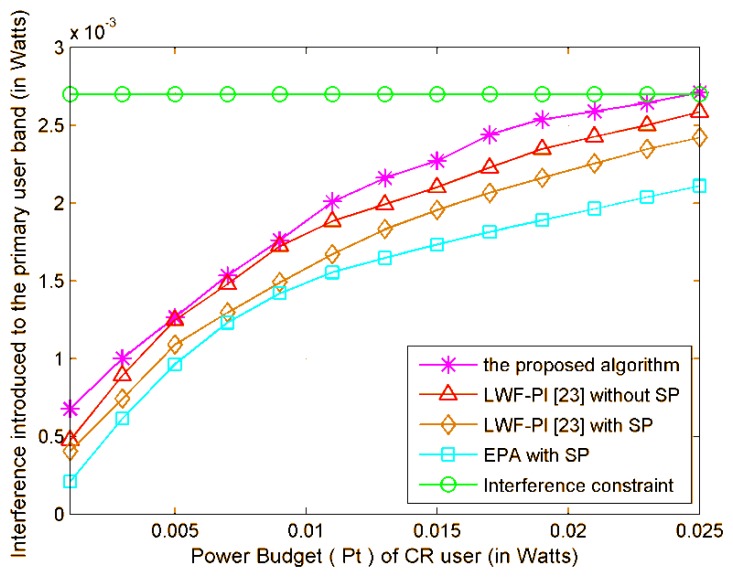
Power Budget *versus* interference introduced to PU bands.

As shown in Figure 4, the achievable maximum CR system transmitted data rate is plotted *versus* the power budget *P_t_*. The upper curve denotes the transmitted data rate by proposed subcarrier-pair based resource allocation algorithm with full CSI. It can be noted that the proposed subcarrier-pair based resource allocation algorithm achieves the highest transmitted data rate under a given total power constraint. The transmitted data rate achieved using the proposed algorithm is the highest among that using LWF-PI-with-SP, Optimal-Scheme-with-SP algorithm, LWF-PI-without-SP algorithm and EPA algorithm. The main reason is that the proposed algorithms can make full use of the entire available interference threshold, while the LWF-PI algorithms can only guarantee that the total interference is under the interference threshold as shown in Figure 5. We observe that only the proposed algorithm outperforms the compared existing resource allocation algorithms. Therefore, we can conclude that the proposed subcarrier-pair based resource allocation algorithm makes valuable contribution to system transmitted data rate.

### 4.2. The Fairness Index Obtained under Resource Allocation Algorithms

**Figure 6.**
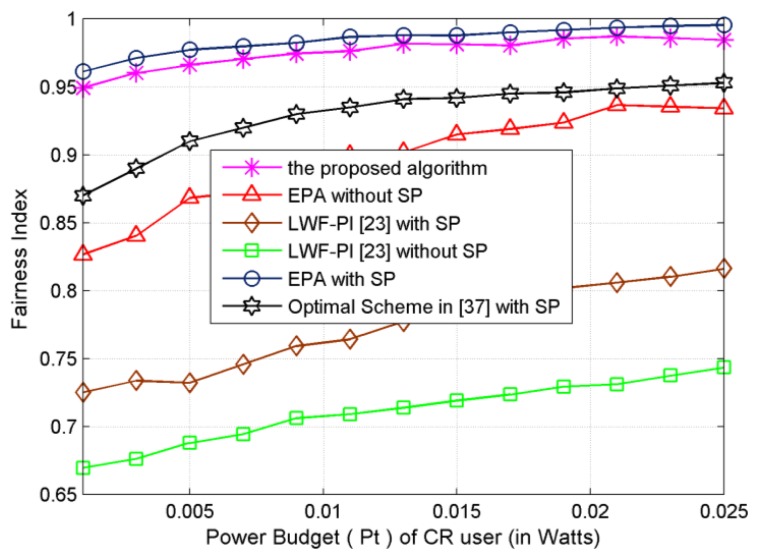
Power Budget *versus* Fairness index.

Both the proposed resource allocation algorithm and EPA-with-SP algorithm exhibit the best fairness performance and LWF-PI-without-SP algorithm shows the least fairness as shown in Figure 6. Moreover, the fairness loss of EPA-with-SP allocation algorithm compared to the proposed subcarrier-pair based resource allocation algorithm is acceptable. In the LWF-PI-without-SP algorithm, LWF-PI-with-SP algorithm and Optimal-Scheme-with-SP algorithm, most power and subcarrier will be assigned to SUs with good channel conditions to improve system efficiency. Unlike the proposed subcarrier-pair based resource allocation algorithm and EPA schemes, these two schemes achieve significant performance improvement to ensure fairness among SUs. The fairness obtained by EPA-with-SP effectively approaches to 0.98. Due to multiuser diversity, the fairness attained by the proposed algorithm is above 0.95, while the achievable system transmitted data rate is higher than that of LWF-PI and EPA-without-SP algorithms.

### 4.3. The Transmitted Data Rate of Each SU for the Resource Allocation Schemes

**Figure 7.**
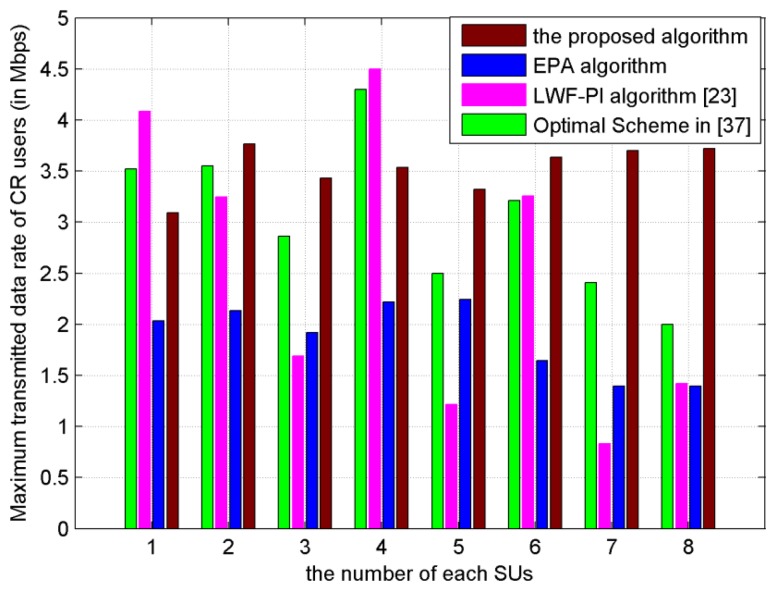
Maximum transmitted data rate *versus* each SU (The power budget *P_t_* is 20 × 10^−3^ W).

As shown in Figure 7, the achievable transmitted data rate of each SU is plotted. The transmitted data rate of each SU, under the proposed subcarrier-pair based resource allocation algorithm, is the most balanced and stable among EPA, LWF-PI and Optimal Scheme [37]. Especially under the LWF-PI algorithm and Optimal Scheme [37], some SUs with bad channel conditions have lower transmitted data rates, which would result in unsuccessful communication with destination node. We can conclude that the proposed subcarrier-pair based resource allocation algorithm makes valuable contribution to balance transmitted data rates among SUs.

## 4.4. The System Transmitted Data Rate Obtained under Full CSI and Partial CSI

**Figure 8.**
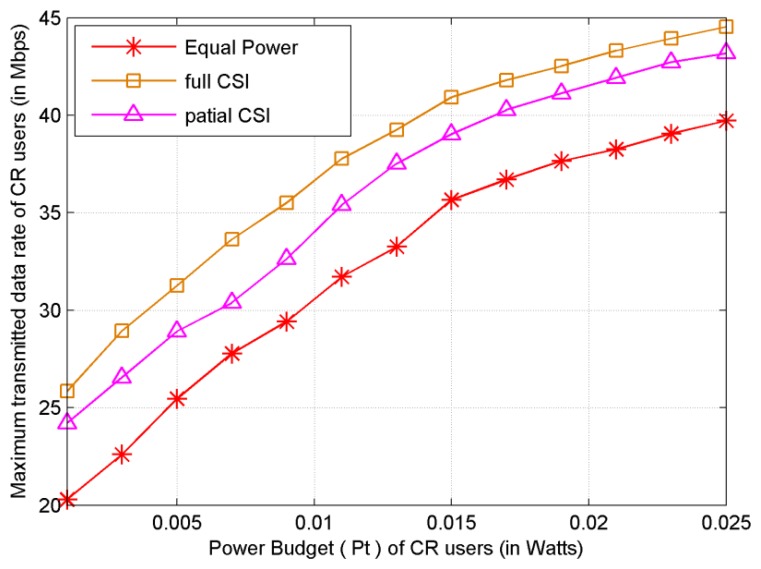
Power Budget *versus* sum transmitted data rate.

As shown in Figure 8, the performance of proposed subcarrier-pair based resource allocation algorithm is presented for the case that each SU has no CSI of non-adjacent link. It is shown that the system transmitted data rate decreases due to limited CSI compared to that of full CSI scenario. However, this scheme does not require the full CSI of non-adjacent link at each cooperative SU. Moreover, we show that the transmitted data rate of the proposed subcarrier-pair based resource allocation algorithm with partial CSI significantly outperforms that of EPA. The transmitted data rate is close to that of subcarrier-pair based resource allocation algorithm with full CSI.

### 4.5. The Transmitted Data Rate Obtained by SUs under Different Distance

**Figure 9.**
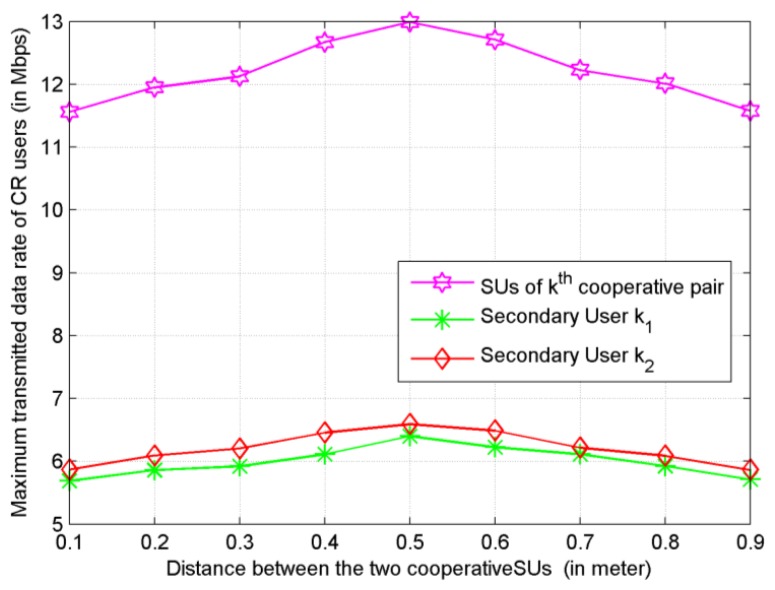
Maximum transmitted data rate *versus* distance between two cooperative SUs.

Last but not least, we have also studied how the distance between cooperative SUs impacts on the system performance. Under the proposed subcarrier-pair based resource allocation algorithm with proportional fairness, Figure 9 shows that the system has comparatively better performance when *d* ∈ [0.4, 0.6] This result provides a guideline for grouping and partner selection in user cooperative networks.

## 5. Conclusions

In this paper, we have developed a novel subcarrier-pair based resource allocation algorithm that maximizes the transmission data rate while the interference introduced to the PUs remains within a given limit. Using the proportional fairness as the optimized objective function, we can improve proportional fairness of resource allocation and achieve substantial transmitted data rate gains. The sum of power constraint for the source and relay nodes are considered. Moreover, we extended the analysis to the case that the CSI of nonadjacent link is not available at cooperative SUs, and found that even in this case the proposed schemes perform better than the classical schemes.

Compared to the existing resource allocation algorithms which are introduced in the paper, our algorithm considers that each secondary relay user has its own data to be transmitted. Simulation results have shown that, either in improving the system throughput or in improving fairness of resource allocation, the proposed subcarrier-pair based resource allocation algorithm offers the best performance conditions among several existing compared resource allocation algorithms under various power budgets, while keeping the interference introduced to PU bands below a specified threshold. Besides, the transmitted data rate of proposed subcarrier-pair based resource allocation algorithm obviously outperforms that of EPA scheme when only partial CSI can be obtained by SUs. The contribution by taking full advantage of the statistical non-adjacent links channel information is demonstrated clearly in the simulation results. In addition, we notice that the system efficiency loss of partial CSI scheme compared to that of full CSI scheme is acceptable.

## Figures and Tables

**Table 1.** Table of Symbols.

**Symbol**	**Definition**
*K*	Number of cooperative partners in the CR network
*N*	Number of subcarriers
Δ*f*	Bandwidth of a subcarrier
*Ts*	Length of a slot
$P_{k1,1}^{n,\text{SP}1(n)}$	Transmission power of *k*1^th^ SU on subcarrier *n* in the first frame
$P_{k1,2}^{n,\text{SP}2(n)}$	Transmission power of *k*1^th^ SU on subcarrier *n* in the second frame
$P_{k2,1}^{n,\text{SP}1(n)}$	Transmission power of *k*2^th^ SU on subcarrier *n* in the first frame
$P_{k2,2}^{n,\text{SP}2(n)}$	Transmission power of *k*2^th^ SU on subcarrier *n* in the second frame
$h_{ki,kj}^{ss,n}(i\neq j)$	the channel gain of the communication link from the *ki*th SU to the *kj* th SU user on the *n*th subcarrier
$h_{ki,0}^{ss,n}$	the channel gain of the communication link from the *ki*th SU to AP on the *n*th subcarrier
$h_{ki,pl}^{sp,n}$	the channel gain of the interference link from *l*th PU to *ki*th SU user receiver on the *n*th subcarrier
$h_{pl,ki}^{ps,n}$	the channel gain of the interference link from *l*th PU to *ki*th SU user receiver on the *n*th subcarrier
$h_{pl,0}^{ps,n}$	the channel gain of the interference link from *l*th PU to AP receiver on the *n*th subcarrier
$\left\{z_{k1}^{\left(i\right)},z_{k2}^{\left(i\right)},z_{k0}^{\left(i\right)},i=1,2,3,4\right\}$	the additive noises at the corresponding node
$\left\{\vartheta_{k1}^{\left(i\right)},\vartheta_{k2}^{\left(i\right)},\vartheta_{k0}^{\left(i\right)},i=1,2,3,4\right\}$	the interference introduced by the PUs into corresponding node

**Table 2.** Power allocation scheme for *k*th cooperative partner on subcarrier *n*.

	**T1 in First Frame**	**T2 in First Frame**	**T1 in Second Frame**	**T2 in Second Frame**
SU*_k1_*	$P_{k1,1}^{n,\text{SP}1(n)}$	0	0	$P_{k2,1}^{n,\text{SP}2(n)}$
SU*_k2_*	0	$P_{k2,2}^{n,\text{SP}1(n)}$	$P_{k1,2}^{n,\text{SP}2(n)}$	0

**Table 3.** Simulation Parameters.

**Parameter**	**Value**
Number of cooperative partners *K*	4
Number of PUs *L*	2
Number of subcarriers *N*	20
Length of a slot *Ts*	4*u* s
value of amplitude *PP_U_*	10 × 10^−3^ W
*Ith*^(*l*)^ (*l* = 1,2)	2.7 × 10^−3^ W
average channel power gain	10 dB
*△ f*	0.315 MHz
*B_2_*	1 MHz
*B_2_*	2 MHz
